# Risk factors and incidence of central venous access device-related thrombosis in hospitalized children: a systematic review and meta-analysis

**DOI:** 10.1038/s41390-024-03225-0

**Published:** 2024-05-17

**Authors:** Maoling Fu, Quan Yuan, Qiaoyue Yang, Yaqi Yu, Wenshuai Song, Xiuli Qin, Ying Luo, Xiaoju Xiong, Genzhen Yu

**Affiliations:** 1 Department of Nursing, Tongji Hospital, Tongji Medical College, Huazhong University of Science and Technology, Wuhan, China; 2 School of Nursing, Tongji Medical College, Huazhong University of Science and Technology, Wuhan, China

## Abstract

**Background:**

The risk factors for central venous access device-related thrombosis (CRT) in children are not fully understood. We used evidence-based medicine to find the risk factors for CRT by pooling current studies reporting risk factors of CRT, aiming to guide clinical diagnosis and treatment.

**Methods:**

A systematic search of PubMed, Web of Science, Embase, Cochrane Library, Scopus, CNKI, Sinomed, and Wanfang databases was conducted. RevMan 5.4 was employed for data analysis.

**Results:**

The review included 47 studies evaluating 262,587 children with CVAD placement. Qualitative synthesis and quantitative meta-analysis identified D-dimer, location of insertion, type of catheter, number of lumens, catheter indwelling time, and central line-associated bloodstream infection as the most critical risk factors for CRT. Primarily due to observational design, the quality of evidence was regarded as low certainty for these risk factors according to the GRADE approach.

**Conclusion:**

Because fewer high-quality studies are available, larger sample sizes and well-designed prospective studies are still needed to clarify the risk factors affecting CRT. In the future, developing pediatric-specific CRT risk assessment tools is important. Appropriate stratified preventive strategies for CRT according to risk assessment level will help improve clinical efficiency, avoid the occurrence of CRT, and alleviate unnecessary suffering of children.

**Impact:**

- This is the latest systematic review of risk factors and incidence of CRT in children.
- A total of 47 studies involving 262,587 patients were included in our meta-analysis, according to which the pooled prevalence of CRT was 9.1%.
- This study identified several of the most critical risk factors affecting CRT in children, including D-dimer, insertion location, type of catheter, number of lumens, catheter indwelling time, and central line-associated bloodstream infection (CLABSI).

## Introduction

Central venous access device (CVAD) is an infusion device inserted through different parts to make the tip of the catheter to the vena cava. In the clinic, CVAD is mainly divided into the following four categories: tunneled central venous catheter (CVC), nontunneled CVC, peripherally inserted central catheter (PICC), and totally implantable venous access port (TIVAP).^1^ Pediatric patients often require stable, multifunctional, and comfortable long-term vascular access due to factors such as poor puncture cooperation, small vessel diameter, poor peripheral venous visibility and tolerance, high water content in the body leading to easy dehydration, and easy changes in condition after diseases.^2^ The application of CVAD can significantly reduce the frequency of venipuncture, relieve the stimulation of drugs on the venous blood vessels, alleviate the pain and fear of the children, improve their medication compliance, ensure the effectiveness of intravenous infusion, and improve the quality of disease treatment.^3–5^ Therefore, CVAD is widely used in pediatric clinics and has become an indispensable aspect of complex medical care for children with severe and chronic diseases.

Although CVAD has become an important tool in the pediatric treatment and nursing process, there are also risks of complications related to it, including CVAD-related thrombosis (CRT), phlebitis, fluid and blood leakage at the puncture point, catheter displacement, catheter obstruction, central line-associated bloodstream infection (CLABSI) and so on.^6,7^ Among these, CRT is one of the most common and serious complications. The prevalence of CRT in children varies significantly by country, age, disease, and medical institution, ranging from 2 to 81%,^4,8–10^ while in Chinese children without prophylactic treatment ranges from 20 to 66%.^11,12^ CRT has no obvious clinical symptoms in the early stage, but it may still cause serious side effects, not only increasing the patient pain and medical costs but also delaying treatment timing, affecting prognosis and quality of life, and in severe cases, may even lead to thromboembolism, endangering life.^13–15^

Identifying risk factors and incidence of CRT facilitates clinical practitioners in the early identification of high-risk patients, designing specific preventive strategies, treatment regimens, and management plans, thereby effectively reducing the incidence of CRT in hospitalized children and alleviating unnecessary patient suffering. However, most current research on CRT involves only small-scale groups in isolated nursing units or specific disease types. To date, no up-to-date systematic review provides pooled estimates of the risk factors and prevalence of CRT in children. Therefore, this study had a dual purpose: 1. to explore potential risk factors for CRT in children and to determine a pooled level of CRT prevalence; and 2. to provide evidence-based recommendations to improve the recognition, control, and treatment of CRT in children, as well as better nursing management for CRT.

## Methods

This review was conducted following the Preferred Reporting Items for Systematic Reviews and Meta-Analyses (PRISMA) guidelines.^16^ The detailed research protocol can be accessed on the PROSPERO website (registration number: CRD42023421353).

### Search strategy

Eight electronic databases were utilized to conduct a thorough literature search: PubMed, Web of Science, Embase, Cochrane Library, Scopus, China National Knowledge Infrastructure (CNKI), Sinomed, and Wanfang. The search in these databases was conducted from the earliest records available up to January 31st, 2024. The search strategy used a combination of Mesh terms and free words. The following Mesh terms and free words were mainly used: “child,” “children,” “adolescent,” “infant,” “pediatrics,” “central venous access device-related thrombosis,” “CRT,” “catheter-related thrombosis,” “catheter-related venous thrombosis,” “CVC-related thrombosis,” “risk factors,” “protective factors,” “predictors,” “causality,” “influencing factors”. The full search strategy for each database is available in the Supplementary Materials. In addition, we screened the reference lists of all included studies for relevant studies that met the criteria. Grey literature was searched as well. Some authors were contacted through email to gather more information or clarify any uncertainties.

### Inclusion criteria

- The study population was hospitalized children aged ≤18 years.
- The primary research objective was to explore the risk factors for CRT.
- The study results have at least one statistically significant predictor.
- Case-control studies or cohort studies.
- Published in English or Chinese.

### Exclusion criteria

- Catheter-related infection, catheter dysfunction, or other catheter complications as the primary outcome indicators.
- Repeated published research.
- Case reports, study designs, or clinical trials.
- Reviews, editorials, letters, and conference abstracts.
- In vitro or animal research.
- Data were incomplete and could not be extracted.
- Unable to find the original article.

### Data extraction

Data from each eligible study were independently extracted by two reviewers using a pre-designed data collection form. Any disagreements were resolved by discussions among all authors. Data on the following characteristics were obtained from all included studies (see Supplementary Table S1 for details):

1. Basic information: first author, country, year of publication, study duration, and study design.
2. Demographic characteristics: study population, sample size, number of CRT, and CRT rate.
3. Catheter-related features: catheter type, CRT type, and diagnostic method.
4. Potential risk factors for CRT: odds ratios (OR) or relative risks (RR) values and 95% confidence interval (CI) were extracted for each risk factor. If the study did not provide specific values, it was calculated by constructing a 2 × 2 contingency table.

### Quality assessment

Two reviewers evaluated the quality of each study independently using the Risk of Bias Assessment for Nonrandomized Studies tool,^17^ with any differences settled via group discussion. The tool assessed six domains of risk of bias: participant selection, confounding variables, exposure measurement, blinding of outcome assessment, incomplete outcome data, and selective outcome reporting. If all six domains were rated as low risk, the overall risk of bias for the study was low. The overall risk of bias was moderate if at least one domain was rated as unclear risk, and no domain was rated as high risk, and high if one or more domains were rated as high risk.

To ensure the accuracy of the assessment results, a third reviewer randomly selected five studies to check the data extraction and quality assessment.

### Qualitative synthesis and quantitative meta-analysis

Qualitatively classify each risk factor as definite, likely, unclear, or not a risk factor based on the total number of studies with low and moderate bias risks and the proportion of studies demonstrating positive association (Box 1 in the supplementary material). If a risk factor was reported by more than two studies with low or moderate risk of bias, and the definition and reference range were sufficiently consistent, a quantitative meta-analysis was performed to estimate the combined OR.

Data were analyzed using Revman 5.4 software. In the meta-analysis of risk factors and CRT rate, the generic inverse variance method was applied, which only required effect estimate and standard error (SE).^18^ The SE was obtained by inverse transforming the 95% CI applying the standard normal distribution. Heterogeneity tests were performed on the studies included in the Meta-analysis to examine for the combinability of the results of each independent study. *P* ≥ 0.05 and I-squared (*I*^2^) < 50% considered less heterogeneity between studies and therefore a fixed-effects model was chosen for the analysis, conversely, *P* < 0.05 or *I*^2^ ≥ 50% considered greater heterogeneity, and a random-effects model was chosen.

### Certainty of the evidence

The Grading of Recommendations Assessment, Development, and Evaluation (GRADE) method was used to assess the certainty of the evidence. In this method, observational studies were initially classified as low-quality evidence and then downgraded and upgraded according to five downgrading and three upgrading principles. The 5 downgrading factors included risk of bias, inconsistency, indirectness, imprecision, and publication bias, and the 3 upgrading factors included the magnitude of an effect, dose-response gradient, and effect of plausible residual confounding. Based on these considerations, the overall certainty of each piece of evidence was rated as one of four levels: high, moderate, low, or very low.

## Results

**Fig. 1:**
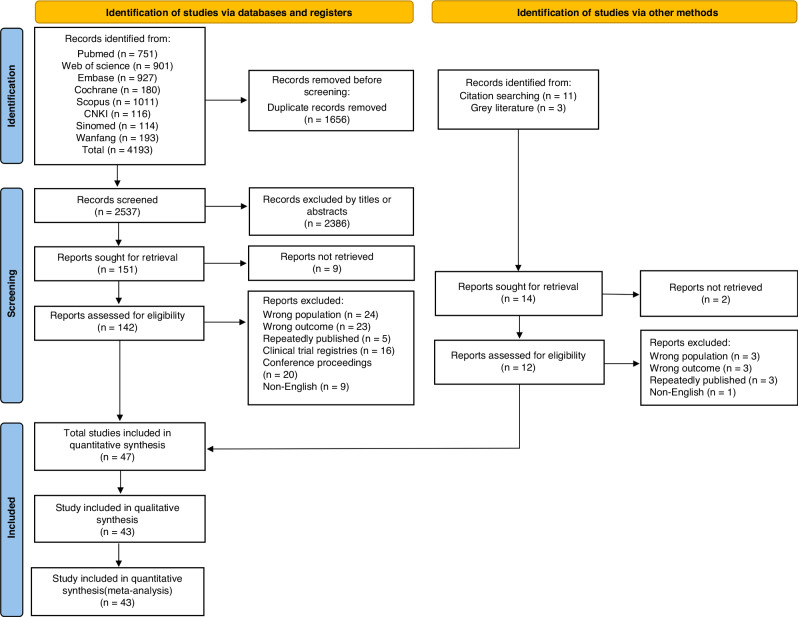
Flow chart of the systematic literature search. Demonstrate the screening and inclusion process for systematic literature search.

The initial search of the databases extracted a total of 4193 articles, of which 1656 were duplicates and removed. The titles and abstracts of the remaining 2537 articles were screened according to the inclusion criteria and 142 were selected for full-text search. After a rigorous eligibility review, 45 articles met the inclusion criteria. In addition, two articles were found to meet the eligibility criteria in a search of the reference lists of the selected articles and grey literature. In the end, a total of 47 articles were included in this review, of which 43 contributed to the qualitative synthesis and quantitative meta-analysis (Fig. 1).

Of the 47 studies, 19 were prospective^4,13,19–35^ and the rest were retrospective,^9,12,36–61^ of which 10 were multicenter^4,9,13,21,23,26–28,49,59^ and 37 were single-center.^12,19,20,22,24,25,29–48,50–58,60,61^ The sample sizes ranged from 47 to 158,299, with the two largest being 71,782^13^ and 158,299,^59^ respectively. In addition, three studies constructed clinical prediction models.^22,28,47^ Table 1 lists the summary characteristics of the included studies.

**Table 1:** Summary characteristics of the 47 studies included in this review

Author	Country of study	Year of publication (data)	Study design	Sample sources	Study population	sample size	Catheter type	CRT rate	CRT type	Diagnostic methods	Potential risk factors
Badheka	America	2021 (2015–2018)	Retrospective cohort	Single-center	Infants younger than 1 year	613	CVC, PICC	64 (10.4%)	Not mention	Ultrasonography	The line tip in IVC, history of thrombosis, history of catheterization, cardiac surgery
Beck	Canada	1998 (1996–1997)	Prospective cohort	Single-center	PICU	93	CVC	17 (18.3%)	DVT	Doppler ultrasound	Cancer, age
Charny	France	2018 (2008–2014)	Retrospective cohort	Single-center	Children with leukemia	192	tunneled CVC, PICC	18 (9.4%)	Not mention	Doppler ultrasound	Type of catheter
Chen	Canada	2016 (2008–2013)	Retrospective cohort	Single-center	Hospitalized children	104	CVC	18 (17.3%)	Not mention	Echocardiography	ALL
Chojnacka	Poland	2022 (2013–2016)	Retrospective cohort	Single-center	NICU	729	CVC	16 (2.2%)	Not mention	Ultrasonography	Asphyxia, infection, catheter indwelling time
Deng	China	2020 (2013–2018)	Retrospective cohort	Single-center	PICU	136	PICC	19 (14.0%)	Not mention	Doppler ultrasound	Type of catheter, D-dimer
Derderian	America	2019 (2013–2016)	Retrospective cohort	Single-center	PICU	2714	CVC	40 (1.5%)	VTE	Doppler ultrasound	Location of insertion
Diamond	America	2018 (2015–2017)	Retrospective cohort	Single-center	Children with active IBD	47	CVC	5 (10.6%)	VTE	Ultrasonography	Anticoagulant thromboprophylaxis
Dubois	Canada	2007 (2004–2005)	Prospective cohort	Single-center	Hospitalized children	214	PICC	20 (9.3%)	DVT	Doppler ultrasound, Venography	Thrombophilia
Faustino	America	2013 (2009–2011)	Prospective cohort	Multicenter	PICU	101	nontunneled CVC	16 (15.8%)	DVT	Doppler ultrasound	Age
Faustino	America	2015 (2012–2013)	Prospective cohort	Multicenter	PICU	85	nontunneled CVC	37 (43.5%)	DVT	Ultrasonography	Thrombophilia
Gnannt	Canada	2018 (2010–2015)	Retrospective cohort	Single-center	Hospitalized children	2180	PICC	92 (4.2%)	DVT, SVT	Ultrasonography	Repetitive PICC insertions in the same arm, PICC material, number of lumens
Gray	America	2012 (2005–2009)	Retrospective cohort	Single-center	Infants younger than 1 year	333	CVC	60 (18.0%)	DVT	Ultrasonography	Location of insertion, type of catheter, number of lumens
Haddad	Canada	2014 (2003–2009)	Retrospective cohort	Single-center	NICU	645	CVC	69 (10.7%)	Not mention	Doppler ultrasound	Birth weight <1000 g, <28 weeks gestation
Jaffray	America	2020 (2013–2018)	Prospective cohort	Multicenter	Hospitalized children	1742	PICC, TL	94 (5.4%)	VTE	Doppler ultrasound	Type of catheter, history of thrombosis, number of lumens, cancer, CLABSI,catheter dysfunction
Jiang	China	2022 (2021–2022)	Prospective cohort	Single-center	Children with congenital heart disease	234	CVC	53 (22.6%)	DVT	Doppler ultrasound	Catheter indwelling time, D-dimer, fibrinogen, days of sedation, vasoactive drugs, pediatric critical illness score,
Jones	Australia	2019 (NA)	Prospective cohort	Single-center	PICU	146	CVC	32 (21.9%)	Not mention	Ultrasonography	Cardiac arrest, location of insertion, hypotension
Kim	Korea	2022 (2018–2020)	Prospective cohort	Single-center	Pediatric surgical patients	80	CVC	31 (38.8%)	internal jugular vein thrombosis	Ultrasonography	Difficult insertion, anesthesia time, blood products, length of hospital admission
Lambert	America	2019 (2013–2015)	Retrospective cohort	Single-center	NICU	766	PICC,UVC,UAC,tunneled long-term catheter	17 (2.2%)	Not mention	Ultrasonography	Catheter size, location of insertion, cholestasis
Li	China	2021 (2015–2018)	Retrospective cohort	Single-center	ICU	1830	CVC	282 (15.4%)	DVT	Doppler ultrasound, computed tomography	Sex, age, ICU admission, catheter size, catheter indwelling time, surgery, infection, cardiac disease
Li	China	2022 (2019–2020)	Retrospective cohort	Single-center	Hospitalized children	594	CVC	158 (26.6%)	Not mention	Doppler ultrasound	Age, hypertonic liquid, catheter placing personnel, insertion length, D-dimer, limb exercises
Longo	Argentina	2021 (2015–2016)	Prospective cohort	Single-center	NICU	264	CVC, PICC, UVC	22 (8.3%)	DVT	Doppler ultrasound, phlebography, echocardiogram	Cardiovascular surgery, NON-PICC
Lovett	America	2023 (2015–2021)	Retrospective cohort	Single-center	Children with sTBI	77	CVC, PICC	23 (29.9%)	DVT	Ultrasonography	Mechanical ventilation duration
MacLean	Canada	2018 (2000–2015)	Retrospective cohort	Multicenter	Pediatric oncology patients	546	CVC	28 (5.1%)	VTE	Radiological imaging	Tissue plasminogen activator
Male	Austria	2003 (1997–1999)	Prospective cohort	Multicenter	Children with leukemia	85	CVL	29 (34.1%)	VTE	Bilateral venography, ultrasound, MRI, echocardiography	Side of insertion, location of insertion, insertion technique
Male	Austria	2005 (1998–1999)	Prospective cohort	Multicenter	Hospitalized children	158	CVL	21 (13.3%)	VTE	Venography, ultrasonography	Location of insertion
Marquez	America	2016 (2009–2013)	Prospective cohort	Multicenter	PICU	175	nontunneled CVC	53 (30.3%)	DVT	Ultrasonography	Age, blood products, surgery, location of insertion, risk of mortality (PIM2 score)
McLaughlin	America	2019 (2006–2016)	Retrospective cohort	Multicenter	Pediatric trauma patients	209	CVC	13 (6.2%)	DVT	Ultrasonography, computed tomography, MRI, venography	Location of insertion
Menéndez	Spain	2016 (2012–2015)	Prospective cohort	Single-center	Hospitalized children	212	PICC	88 (41.5%)	DVT, SVT	Doppler ultrasound	Catheter to vein ratio,procedure time, location of insertion, SVT, catheter indwelling time,non-optimal tip location
Noonan	America	2018 (2012–2016)	Retrospective cohort	Single-center	Hospitalized children	2709	PICC, CVC	70 (2.6%)	VTE	Radiographic imaging	Type of catheter, side of insertion
Onyeama	America	2018 (2012–2016)	Retrospective cohort	Single-center	Children with hematologic malignancies	198	CVC	18 (9.1%)	VTE	Doppler ultrasound, MRV, computed tomography	Age, type of catheter, sepsis, tissue plasminogen activator
Östlund	Sweden	2019 (2015–2016)	Prospective cohort	Single-center	Hospitalized children	211	nontunneled CVC	64 (30.3%)	Not mention	Doppler ultrasound	Location of insertion, number of lumens, sex
Patel	America	2020 (2010–2015)	Prospective cohort	Multicenter	PICU	71782	CVAD	399 (0.6%)	VTE	Not mention	Type of catheter, line presence at admission, geographic location of line placement, location of insertion, freestanding children’s hospital
Pei	China	2016 (2011–2015)	Retrospective cohort	Single-center	PICU	105	CVC	7 (6.7%)	DVT	Ultrasonography	TPN, hypertonic liquid
Rooden	Netherlands	2005 (2000–2002)	Prospective cohort	Single-center	Children in hematology	105	CVC	13 (12.4%)	Not mention	Doppler ultrasound or venography	CLABSI
Shah	America	2015 (2010–2012)	Retrospective cohort	Single-center	Hospitalized children	3733	CVL	62 (1.7%)	Not mention	Doppler ultrasound, echocardiography	Type of catheter, location of insertion, geographic location of line placement
Shin	America	2017 (2010–2013)	Retrospective cohort	Single-center	Hospitalized children	1100	PICC	292 (26.3%)	Not mention	Doppler ultrasounds, contrast venograms, MRA	History of catheterization
Smitherman	America	2015 (2009–2012)	Retrospective cohort	Single-center	Noncritically ill Children	815	PICC, tunneled, nontunneled, and port	36 (4.4%)	DVT, SVT	Doppler ultrasound, venogram, MRV, echocardiogram	Age, dialysis, diagnosis of IBD or SBS
Sol	Netherlands	2015 (2007–2009)	Prospective cohort	Single-center	PICU	134	femoral CVC	13 (9.7%)	Not mention	Ultrasonography	CLABSI
Steen	America	2019 (2017)	Retrospective cohort	Single-center	CICU	747	nontunneled CVC,UVC	57 (7.6%)	DVT	Ultrasonography	Number of catheters, age, LCOS, sepsis, CLABSI, cardiac catheterization, cardiac surgery
Tran	America	2018 (2009–2014)	Retrospective cohort	Multicenter	PICU	158299	CVC	1602 (1.0%)	VTE	Not mention	Age, mechanical ventilation, surgery, cardiac catheterization, type of catheter, ECMO, neurological disease, autoimmune disease, cancer
Verheij	Netherlands	2018 (2006–2013)	Retrospective cohort	Single-center	NICU	552	FVC, UVC, PICC	14 (2.5%)	Not mention	Doppler ultrasound	Type of catheter
Wang	China	2021 (2015–2019)	Retrospective cohort	Single-center	Children with leukemia	908	PICC	19 (2.1%)	VTE	Doppler ultrasound	D-dimer
Wei	China	2017 (2014)	Prospective cohort	Single-center	Children with leukemia	116	PICC	33 (28.4%)	Not mention	Ultrasonography	Side of insertion, D-dimer
Wisecup	America	2015 (2007–2012)	Retrospective cohort	Single-center	Hospitalized children	2388	CVL	125 (5.2%)	VTE	Ultrasonography	Congenital heart defect, TPN
Zeng	China	2020 (2016)	Prospective cohort	Single-center	PICU	338	CVC	87 (25.7%)	DVT	Doppler ultrasound	Type of catheter, glucocorticoid, TPN
Zhu	China	2022 (2014–2021)	Retrospective cohort	Single-center	NICU	3043	PICC	7 (0.23%)	Not mention	Ultrasonography	Mothers’ use of anticoagulants, cardiac insufficiency, TTTS-donor

*CRT* central venous access device-related thrombosis, *CVC* central venous catheter, *PICC* peripherally inserted central catheter, *IVC* inferior vena cava, *PICU* pediatric intensive care unit, *DVT* deep vein thrombosis, *ALL* acute lymphoblastic leukemia, *NICU* neonatal intensive care unit, *VTE* venous thromboembolism, *IBD* inflammatory bowel disease, *PIM2*: pediatric index of mortality 2, *SVT* superficial venous thrombosis, *TL* tunneled line, *CLABSI* central line-associated bloodstream infection, *UVC* umbilical venous catheter, *UAC* umbilical arterial catheters, *sTBI* severe traumatic brain injury, *CVL* central venous lines, *MRI* magnetic resonance imaging, *MRV* magnetic resonance venography, *TPN* total parenteral nutrition, *MRA* magnetic resonance angiograms, *SBS* short bowel syndrome, *LCOS* low cardiac output syndrome, *ECMO* extracorporeal membrane oxygenation, *FVC* femoral venous catheter, *TTTS* twin-to-twin transfusion syndrome.

### Study populations and CRT rates in included studies

These studies investigated a series of hospitalized children of different ages and departments, of which 12 studies with all hospitalized children as the study population, 12 studies with PICU hospitalized children as the study population, six studies with NICU hospitalized children as the study population, one study with all ICU hospitalized children as the study population, four studies with leukemia children as the study population, two studies with infants under 1-year-old as the study population, and the other ten studies with children with a specific disease as the study population.

The combined CRT rate was 9.1% (95% *CI*: 5.7–14.5%) with a high degree of heterogeneity (*I*^2^ = 100%). The combined CRT rate was 11.5% (95% *CI*: 5.7–23.1%; *I*^2^ = 99%) in both male and female children. The frequency of CRT in PICU and NICU was available from 13 articles with 234,464 children and 7 articles with 6093 infants, which combined CRT rates were 10.7% (95% *CI*: 3.8–23.7%; *I*^2^ = 100%), 2.9% (95% *CI*: 1.0–6.5%; *I*^2^ = 96%), respectively. The combined CRT rate of children with leukemia was 13.0% (95% *CI*: 2.9–38.3%; *I*^2^ = 98%) (Supplementary Material Figs. S1–6)

### Quality of the CRT studies

**Fig. 2:**
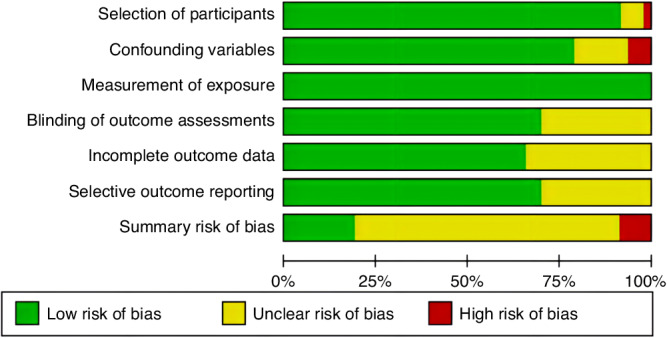
Summary of risk of bias in the included studies. A summary presentation of the assessment results of risk of bias for the 47 studies.

The methodological quality of the included studies varied (Fig. 2 and Supplementary Material Fig. S7). Nine studies had a low overall risk of bias, as all six domains were categorized as low risk. Four studies had a high overall risk of bias, three of which were associated with confounding variables and one to participant selection. The remaining 34 studies had a moderate overall risk of bias, with at least one of the six domains having an unclear risk.

### Risk factors of CRT in included studies

The 47 included studies reported 61 statistically significant risk factors for CRT (Table 1). These factors were classified into three categories: patient-related risk factors (37.7%, 23/61); CVAD-related risk factors (34.4%, 21/61), and treatment-related risk factors (27.9%, 17/61).

Based on the qualitative synthesis, six variables were considered to be definite risk factors for CRT, including D-dimer, location of insertion, type of catheter, number of lumens, catheter indwelling time, and CLABSI. Eleven variables were considered likely associated with CRT, including gastrointestinal diseases, history of catheterization, thrombophilia, geographic location of line placement, catheter dysfunction, number of catheters, insertion length (cm), catheter to vein ratio, dialysis, hypertonic liquid, and cardiac catheterization. For 42 variables, the relationship with CRT was deemed unclear due to conflicting results from studies assessed as having low and moderate risk of bias, or because they were positively associated in only one study. Additionally, birth weight and gestational age were considered non-risk factors (Table 2).

**Table 2:** Summary of CRT risk factors

Categories	Risk factors
Definite factors (6)	D-dimer, location of insertion, type of catheter, number of lumens, catheter indwelling time and CLABSI
Likely factors (11)	Gastrointestinal diseases, history of catheterization, thrombophilia, geographic location of line placement, catheter dysfunction, number of catheters, insertion length (cm), catheter to vein ratio, dialysis, hypertonic liquid, and cardiac catheterization
Unclear factors (42)	Sex, age, history of thrombosis, infection, sepsis, cancer, cardiovascular disease, neurological disease, autoimmune disease, asphyxia, ECMO, catheter size, side of insertion, non-optimal tip location, difficult insertion, TPN, surgery, mechanical ventilation, mechanical ventilation duration, mechanicalthromboprophylaxis / limb exercises, tissue plasminogen activator, glucocorticoid, vasoactive drugs, blood products, length of hospital admission, fibrinogen, PICC material, catheter placing personnel, insertion technique, the line tip in IVC, repetitive insertions in the same arm, procedure time, line presence at admission, anesthesia time, SVT, ICU admission, days of sedation, pediatric critical illness score, risk of mortality (PIM2 score), mothers’ use of anticoagulants, TTTS-donor, freestanding children’ s hospital
Not a risk factor (2)	Birth weight and gestational age

*CLABSI* central line-associated bloodstream infection, *ECMO* extracorporeal membrane oxygenation, *TPN* total parenteral nutrition, *PICC* peripherally inserted central catheter, *IVC* inferior vena cava, *SVT* superficial venous thrombosis, *ICU* intensive care unit, *PIM2* pediatric index of mortality 2, *TTTS* twin-to-twin transfusion syndrome.

**Fig. 3:**
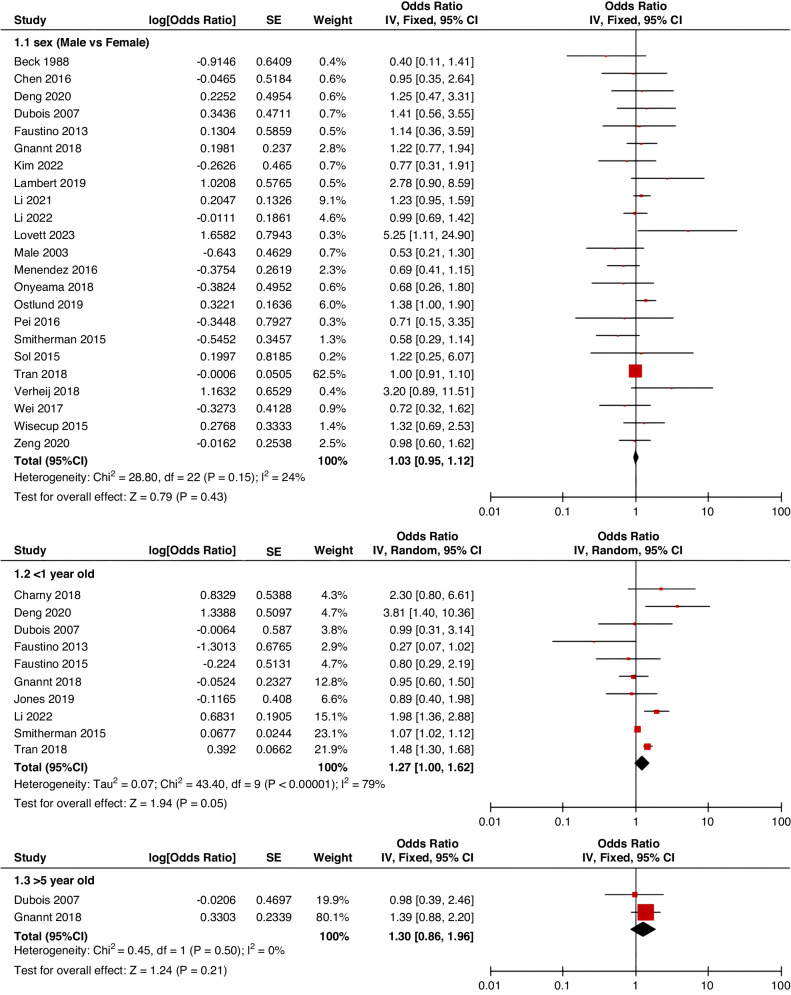

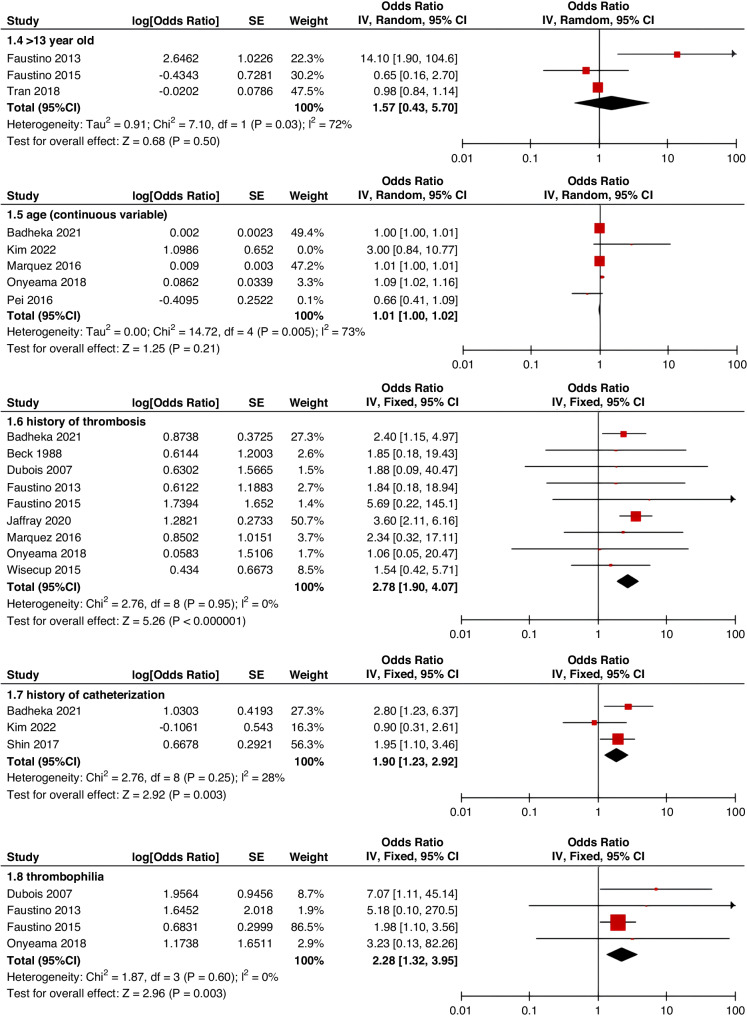

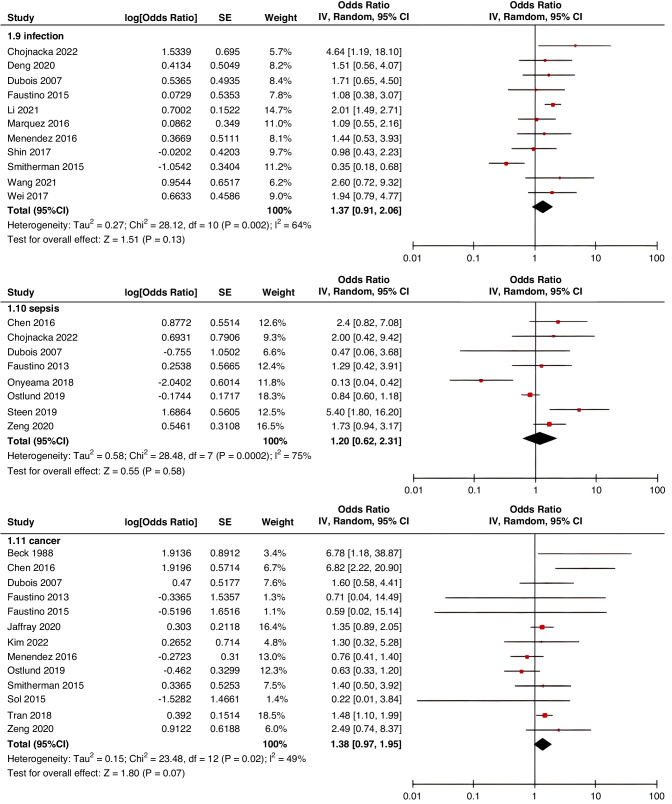

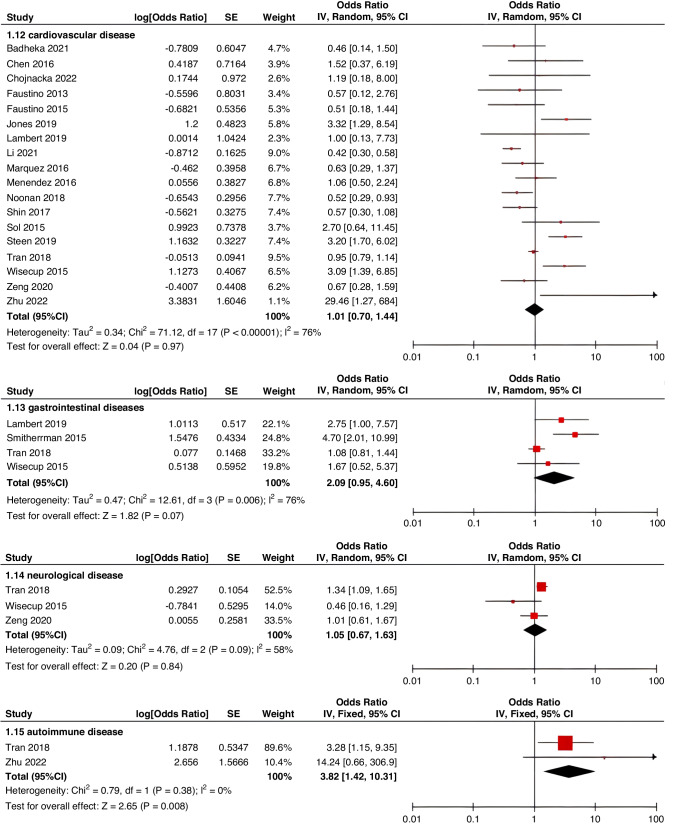

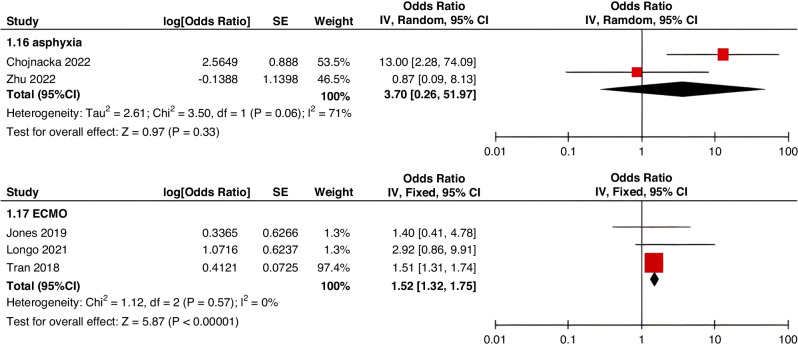
Meta-analysis of patient-related risk factors. Forest plots of odds ratios (OR) that were included in the quantitative meta-analysis and the associated overall OR. For each OR, the size of the red square region is proportional to the corresponding study weight. Diamond shape intervals represent the overall OR. I^2^ represents the fraction of variability among the individual OR that cannot be explained by sampling variability.

**Fig. 4:**
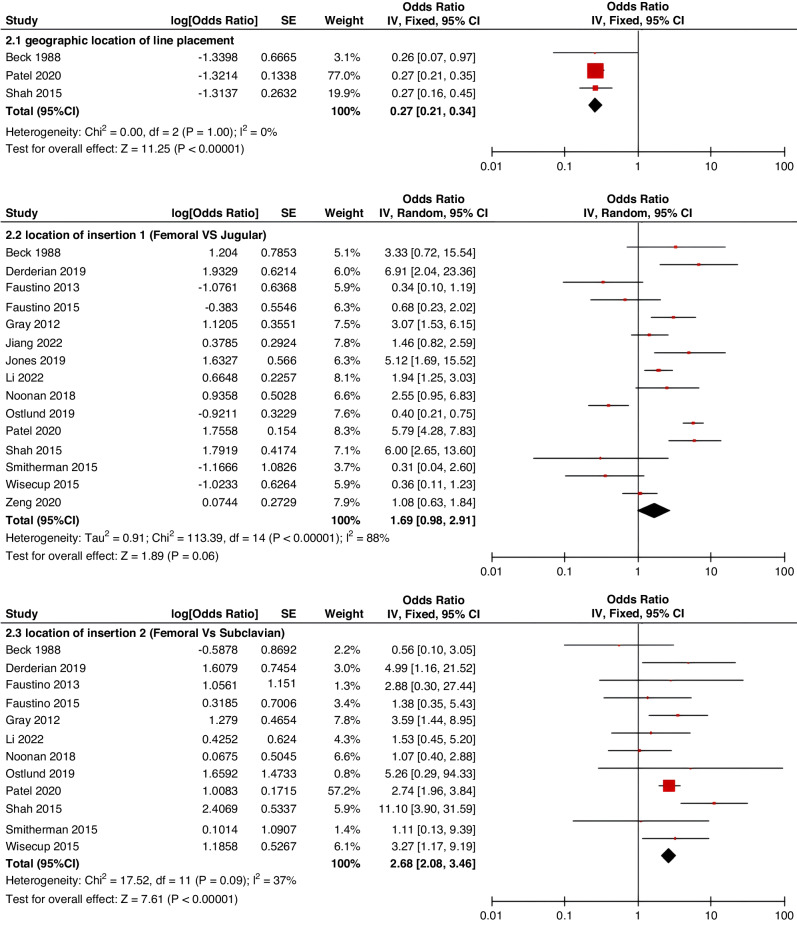

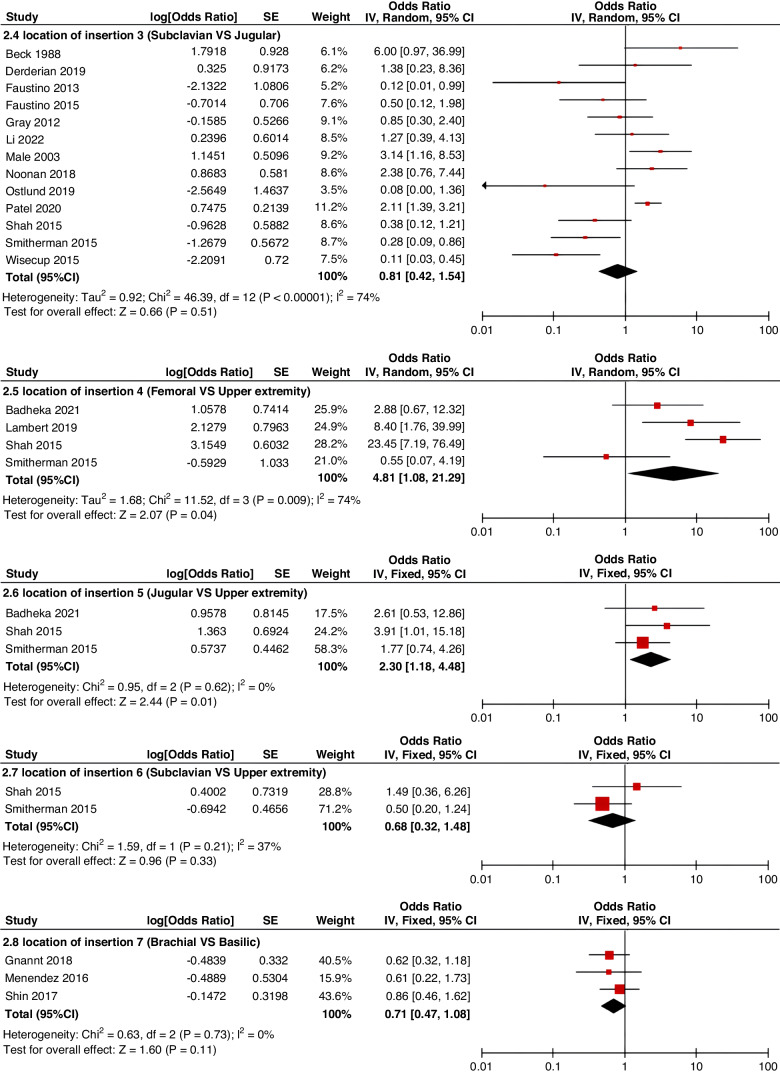

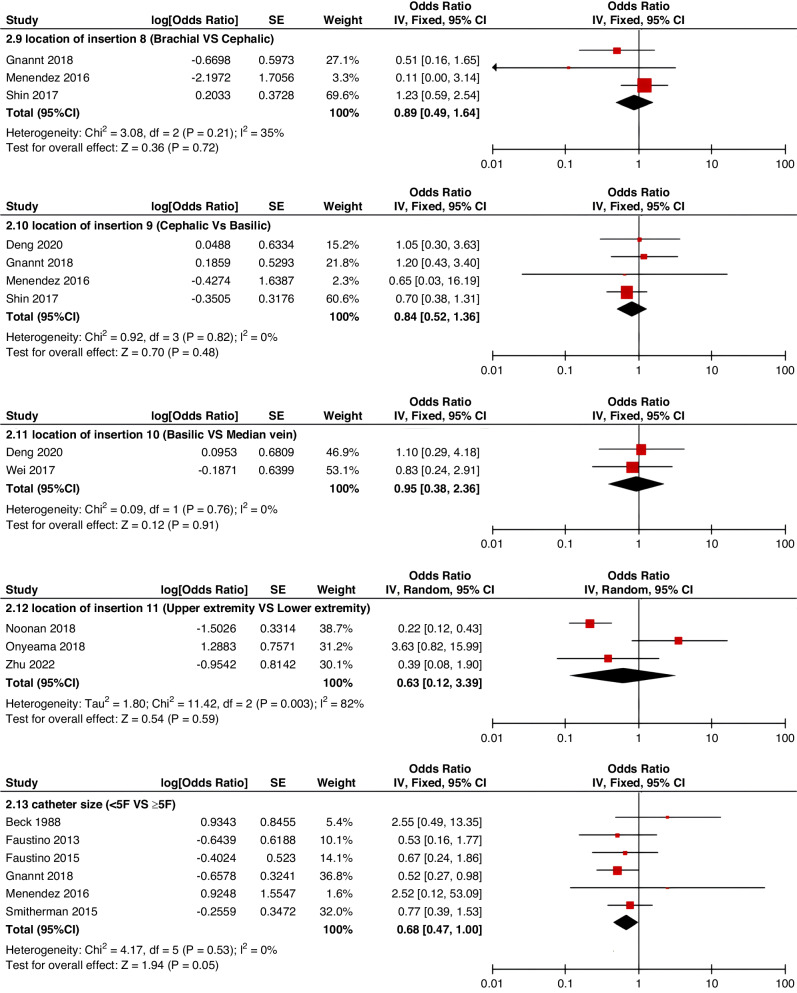

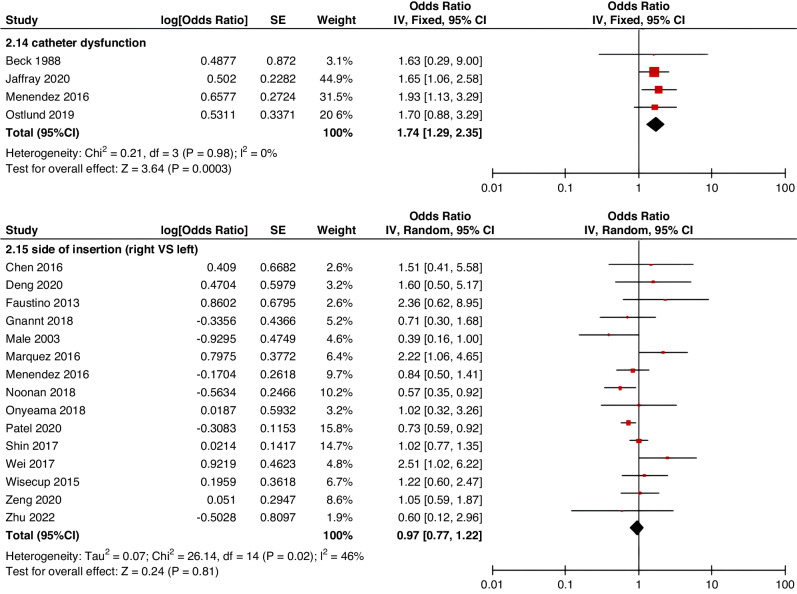
Meta-analysis of CVAD-related risk factors (1). Forest plots of odds ratios (OR) that were included in the quantitative meta-analysis and the associated overall OR. For each OR, the size of the red square region is proportional to the corresponding study weight. Diamond shape intervals represent the overall OR. I^2^ represents the fraction of variability among the individual OR that cannot be explained by sampling variability.

**Fig. 5:**
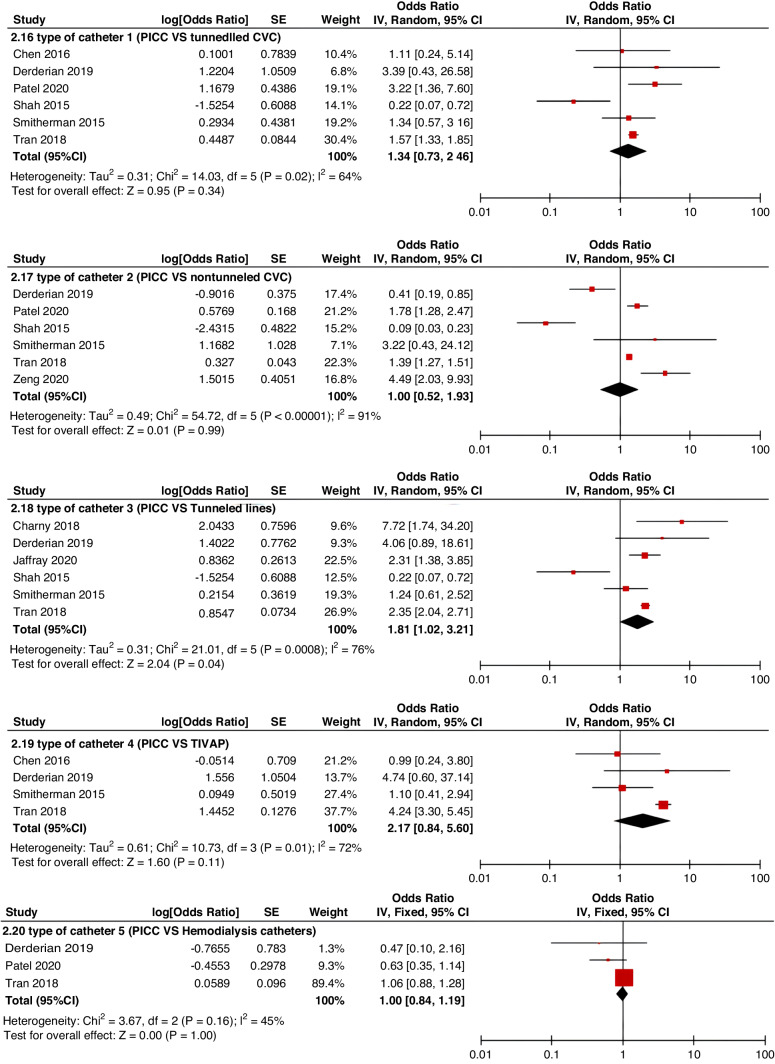

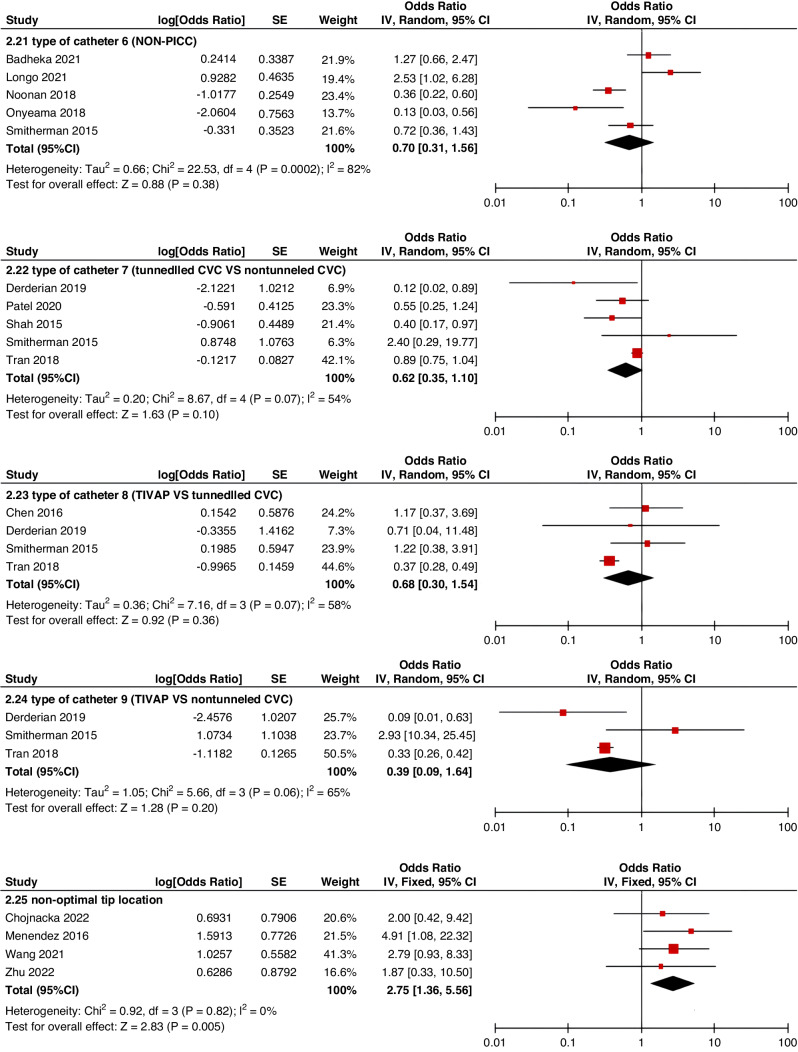

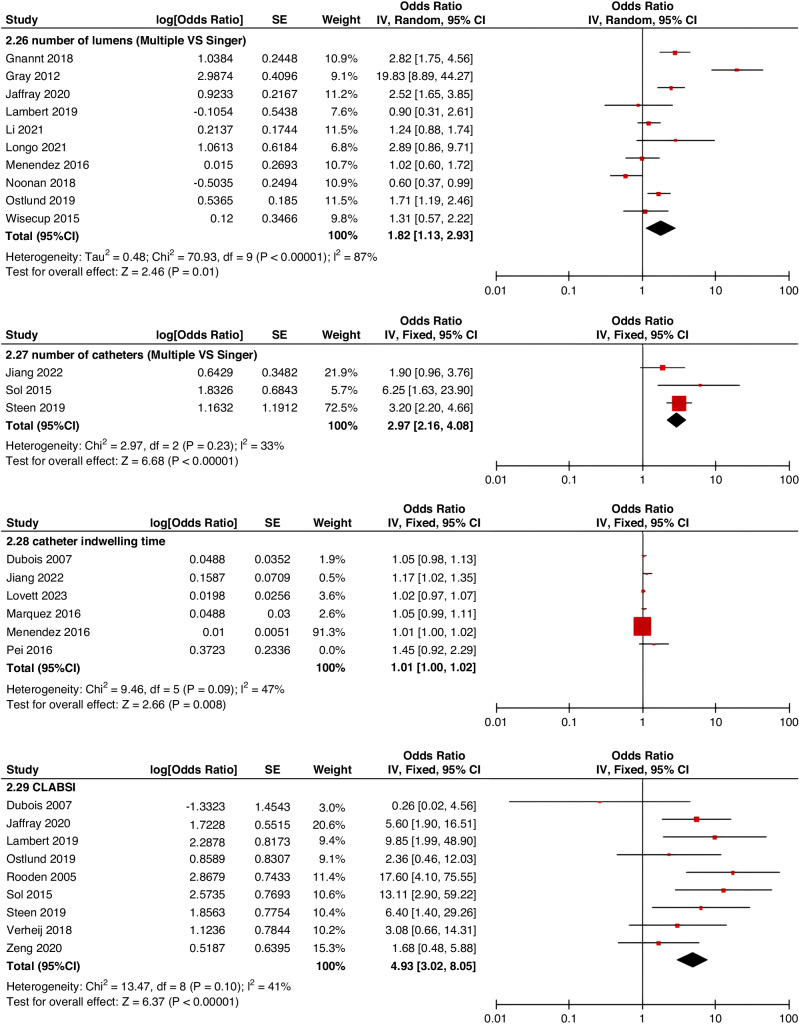

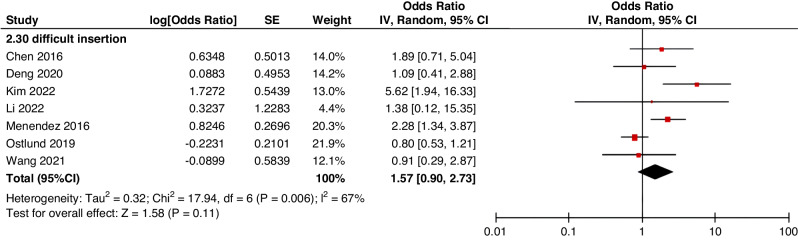
Meta-analysis of CVAD-related risk factors (2). Forest plots of odds ratios (OR) that were included in the quantitative meta-analysis and the associated overall OR. For each OR, the size of the red square region is proportional to the corresponding study weight. Diamond shape intervals represent the overall OR. I^2^ represents the fraction of variability among the individual OR that cannot be explained by sampling variability.

**Fig. 6:**
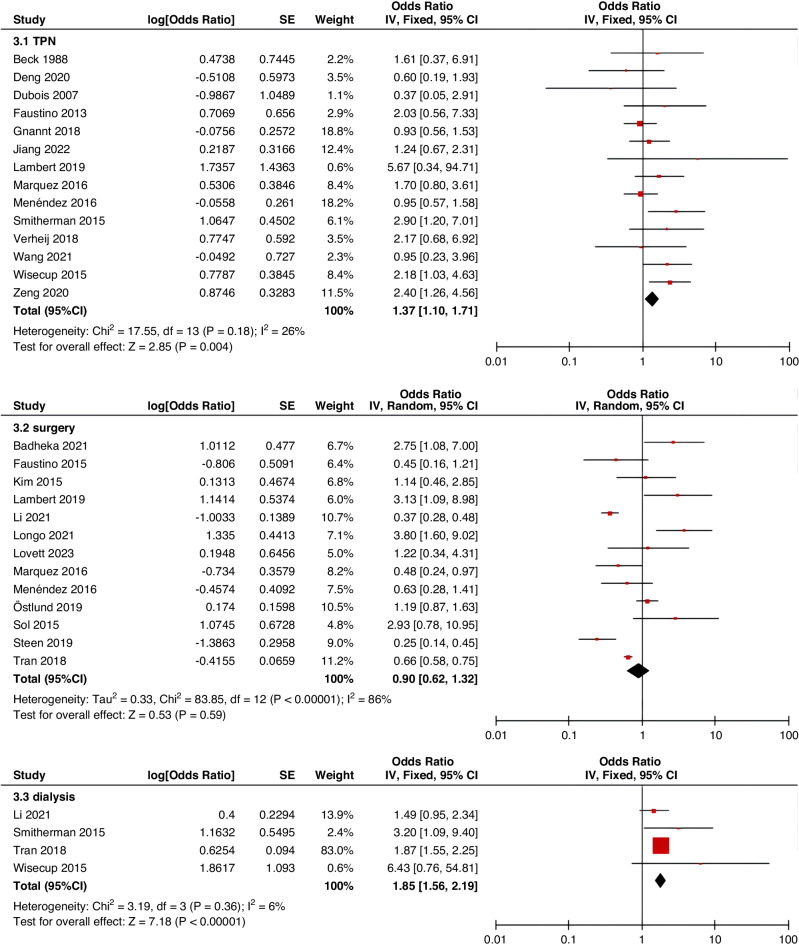

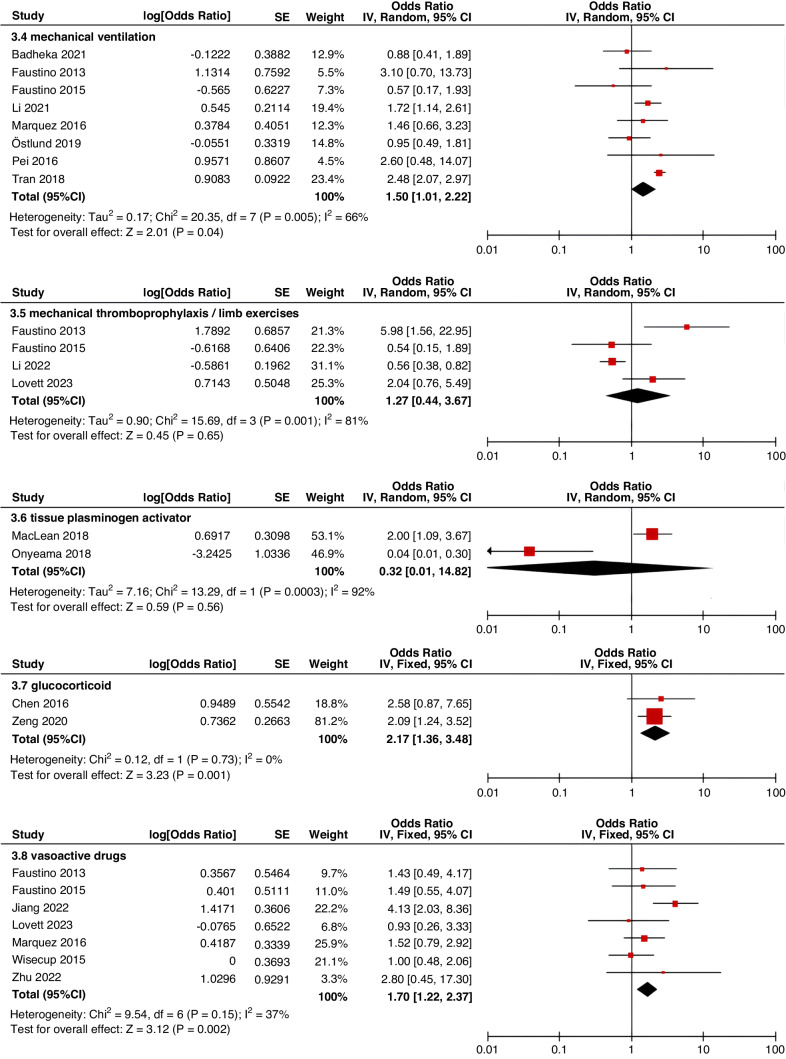

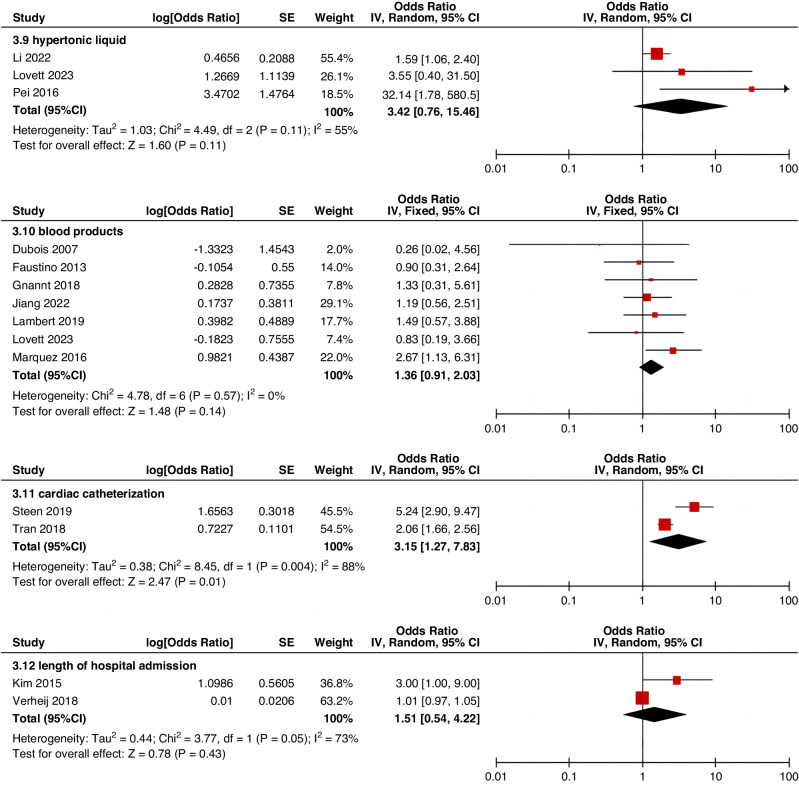
Meta-analysis of treatment-related risk factors. Forest plots of odds ratios (OR) that were included in the quantitative meta-analysis and the associated overall OR. For each OR, the size of the red square region is proportional to the corresponding study weight. Diamond shape intervals represent the overall OR. I^2^ represents the fraction of variability among the individual OR that cannot be explained by sampling variability.

Meta-analyses were implemented for risk factors that were reported by at least two low or moderate risk of bias studies with a consistent definition and reference range (Table 3 and Figs. 3–6).

**Table 3:** The effect size of risk factors for CRT in children

Categories	Potential risk factors	*n*	Effect size			Heterogeneity		Analyzed model
			odds ratio	95% CI	*P*-Value	I^2^ (%)	*P*-Value	
Patient-related risk factors	Sex (Male vs Female)	23	1.03	0.95–1.12	0.43	24	0.15	Fixed
	Age: <1 year old	10	1.27	1.00–1.62	0.05	79	<0.00001	Random
	Age: >5 year old	2	1.30	0.86–1.96	0.21	0	0.50	Fixed
	Age: >13 year old	3	1.57	0.43–5.70	0.50	72	0.03	Random
	Age: continuous variable	5	1.01	1.00–1.02	0.21	73	0.005	Random
	History of thrombosis	9	2.78	1.90–4.07	<0.00001	0	0.95	Fixed
	History of catheterization	3	1.90	1.23–2.92	0.003	28	0.25	Fixed
	Thrombophilia	4	2.28	1.32–3.95	0.003	0	0.60	Fixed
	Infection	11	1.37	0.91–2.06	0.13	64	0.002	Random
	Sepsis	8	1.20	0.62–2.31	0.58	75	0.0002	Random
	Cancer	13	1.38	0.97–1.95	0.07	49	0.02	Random
	Cardiovascular disease	18	1.01	0.70–1.44	0.97	76	<0.00001	Random
	Gastrointestinal diseases	4	2.09	0.95–4.60	0.07	76	0.006	Random
	Neurological disease	3	1.05	0.67–1.63	0.84	58	0.09	Random
	Autoimmune disease	2	3.82	1.42–10.31	0.008	0	0.38	Fixed
	Asphyxia	2	3.70	0.26–51.97	0.33	71	0.06	Random
	ECMO	3	1.52	1.32–1.75	<0.00001	0	0.57	Fixed
CVAD-related risk factors	Geographic location of line placement	3	0.27	0.21–0.34	<0.00001	0	1.00	Fixed
	Location of insertion 1 (Femoral VS Jugular)	15	1.69	0.98–2.91	0.06	88	<0.00001	Random
	Location of insertion 2 (Femoral VS Subclavian)	12	2.68	2.08–3.46	<0.00001	37	0.09	Fixed
	Location of insertion 3 (Subclavian VS Jugular)	13	0.81	0.42–1.54	0.51	74	<0.00001	Random
	Location of insertion 4 (Femoral VS Upper extremity)	4	4.81	1.08–21.29	0.04	74	0.009	Random
	Location of insertion 5 (Jugular VS Upper extremity)	3	2.30	1.18–4.48	0.01	0	0.62	Fixed
	Location of insertion 6 (Subclavian VS Upper extremity)	2	0.68	0.32–1.48	0.33	37	0.21	Fixed
	Location of insertion 7 (Brachial VS Basilic)	3	0.71	0.47–1.08	0.11	0	0.73	Fixed
	Location of insertion 8 (Brachial VS Cephalic)	3	0.89	0.49–1.64	0.72	35	0.21	Fixed
	Location of insertion 9 (Cephalic VS Basilic)	4	0.84	0.52–1.36	0.48	0	0.82	Fixed
	Location of insertion 10 (Basilic VS Median vein)	2	0.95	0.38–2.36	0.91	0	0.76	Fixed
	Location of insertion 11 (Upper extremity VS Lower extremity)	3	0.63	0.12–3.39	0.59	82	0.003	Random
	Catheter size (<5 F VS ≥ 5 F)	6	0.68	0.47–1.00	0.05	0	0.53	Fixed
	Catheter dysfunction	4	1.74	1.29–2.35	0.0003	0	0.98	Fixed
	Side of insertion (right VS left)	15	0.97	0.77–1.22	0.81	46	0.02	Random
	Type of catheter 1 (PICC VS tunnedlled CVC)	6	1.34	0.73–2.46	0.34	64	0.02	Random
	Type of catheter 2 (PICC VS nontunneled CVC)	6	1.00	0.52–1.93	0.99	91	<0.00001	Random
	Type of catheter 3 (PICC VS Tunneled lines)	6	1.81	1.02–3.21	0.04	76	0.0008	Random
	Type of catheter 4 (PICC VS TIVAP)	4	2.17	0.84–5.60	0.11	72	0.01	Random
	Type of catheter 5 (PICC VS Hemodialysis catheters)	3	1.00	0.84–1.19	1.00	45	0.16	Fixed
	Type of catheter 6 (NON-PICC)	5	0.70	0.31–1.56	0.38	82	0.0002	Random
	Type of catheter 7 (tunnedlled CVC VS nontunneled CVC)	5	0.62	0.35–1.10	0.10	54	0.07	Random
	Type of catheter 8 (TIVAP VS tunnedlled CVC)	4	0.68	0.30–1.54	0.36	58	0.07	Random
	Type of catheter 9 (TIVAP VS nontunneled CVC)	3	0.39	0.09–1.64	0.20	65	0.06	Random
	Non-optimal tip location	4	2.75	1.36–5.56	0.005	0	0.82	Fixed
	Number of lumens (Multiple VS Singer)	10	1.82	1.13–2.93	0.01	87	<0.00001	Random
	Number of catheters (Multiple VS Singer)	3	2.97	2.16–4.08	<0.00001	33	0.23	Fixed
	Catheter indwelling time	6	1.01	1.00–1.02	0.008	47	0.09	Fixed
	CLABSI	9	4.93	3.02–8.05	<0.00001	41	0.10	Fixed
	Difficult insertion	7	1.57	0.90–2.73	0.11	67	0.006	Random
Treatment-related risk factors	TPN	14	1.37	1.10–1.71	0.004	26	0.18	Fixed
	Surgery	13	0.90	0.62–1.32	0.59	86	<0.00001	Random
	Dialysis	4	1.85	1.56–2.19	<0.00001	6	0.36	Fixed
	Mechanical ventilation	8	1.50	1.01–2.22	0.04	66	0.005	Random
	Mechanical thromboprophylaxis/limb exercises	4	1.27	0.44–3.67	0.65	81	0.001	Random
	Tissue plasminogen activator	2	0.32	0.01–14.82	0.56	92	0.0003	Random
	Glucocorticoid	2	2.17	1.36–3.48	0.001	0	0.73	Fixed
	Vasoactive drugs	7	1.70	1.22–2.37	0.002	37	0.15	Fixed
	Hypertonic liquid	3	3.42	0.76–15.46	0.11	55	0.11	Random
	Blood products	7	1.36	0.91–2.03	0.14	0	0.57	Fixed
	Cardiac catheterization	2	3.15	1.27–7.83	0.01	88	0.004	Random
	Length of hospital admission	2	1.51	0.54–4.22	0.43	73	0.05	Random

*CRT* central venous access device-related thrombosis, *CI* confidence interval, *I*^*2*^ tested for heterogeneity, *ECMO* extracorporeal membrane oxygenation, *PICC* peripherally inserted central catheter, *CVC* central venous catheter, *TIVAP* totally implantable venous access port, *CLABSI* central line-associated bloodstream infection, *TPN* total parenteral nutrition.

### GRADE assessment of evidence

Supplementary Table S2 shows GRADE assessments for the certainty of evidence. Due to the design of the observational studies, all evidence was initially rated as low certainty. Based on five downgrading and three upgrading principles, 17 pieces of evidence were still rated as low certainty, and the remaining 44 pieces of evidence were downgraded to very low certainty for serious inconsistency and imprecision.

## Discussion

Our study is the latest systematic review of risk factors and the incidence of CRT in hospitalized children. Based on 47 studies included in the current meta-analysis, which involved a total of 262,587 patients, the pooled prevalence of CRT is 9.1%. We conducted a qualitative synthesis analysis of 61 predictive factors and a quantitative meta-analysis of 38 factors, identifying six definite factors, 11 likely factors, and 42 unclear factors associated with CRT. Definite predictors included being of D-dimer, location of insertion, type of catheter, number of lumens, catheter indwelling time and CLABSI. The findings of our systematic review provide the latest comprehensive evidence summary that can inform the early identification of children at risk for CRT and the development of intervention measures to prevent and reduce CRT.

Implantable and temporary medical devices such as CVAD are exposed to blood for weeks to years depending on the type of CVAD in place. Since CVAD is an artificial surface and lacks an endothelial layer that inhibits platelet coagulation and adhesion, it is thought to potentially activate the contact pathways, ultimately leading to thrombosis. Assembly of artificial surface contact systems might be part of the host defense mechanism against foreign substances, but it can lead to kinin and thrombin generation, and complement activation.^62^ This eventually promotes thrombosis and inflammation. The presence of CVAD is the most common risk factor for venous thromboembolism (VTE). CRT accounts for 10% of deep vein thrombosis (DVT) in adults and 50–80% in children.^10,55,63^ The incidence of CRT in hospitalized children has increased significantly by 30–70% over the past 20 years,^64,65^ which may cause serious medical complications besides increasing healthcare expenditures and length of stay.

We discover that a higher level of D-dimer is an independent risk factor for CRT in hospitalized children, consistent with the results of adult studies.^66^ D-dimer is a soluble fibrin degradation product deriving from the plasmin-mediated degradation of cross-linked fibrin that is increased or positive in secondary hyperfibrinolysis, such as hypercoagulable states, disseminated intravascular coagulation, and thrombolytic therapy.^67,68^ Increased D-dimer suggests an association with thrombotic disorders in the body of various origins and an increase in fibrinolytic activity. D-dimer has been extensively investigated for excluding the diagnosis of VTE and is used routinely for this indication.^67,69^ Therefore, for early recognition and to reduce the incidence of CRT, D-dimer levels should be closely monitored before and after catheterization. However, the elevated D-dimer test results cannot fully explain the cause and location of CRT formation and must be analyzed in conjunction with clinical and other test results. Inherited thrombophilia, caused by genetic defects leading to a deficiency or abnormality in associated proteins, including protein C, protein S, antithrombin, the coagulation factor V Leiden mutation, and factor II mutation G20210A,^70^ is considered a potential risk factor for CRT. The prevalence of thrombophilia varies widely among different populations, with a reported prevalence of 10% to 59% in pediatric VTE patients.^71^ Children with gastrointestinal diseases like short bowel syndrome (SBS) and inflammatory bowel disease (IBD) have an increased risk of developing CRT during hospitalization. The precise mechanism behind this association is still uncertain according to current research. It may be attributed to the heightened inflammation levels during catheterization, particularly in patients with active IBD episodes or admissions during surgery, which leads to a period of increased inactivity.^55^ This suggests that delaying placement during the most active period of inflammation may reduce the rate of thrombosis.

A narrative review pointed out that age is one of the most significant risk factors for VTE. In children, CRT shows a bimodal distribution, with the highest incidence rate in infancy and adolescence.^10^ The higher incidence in infancy may be due in part to the smaller diameter of the vein, making insertion difficult and requiring multiple attempts. However, whether age is a risk factor for CRT is still highly controversial. The study by Chojnacka et al. did not find a statistically significant difference,^39^ although a trend toward a similar bimodal distribution was found in the study population. Cancer, cardiovascular disease, sepsis, asphyxia, and neurological diseases are also considered unclear factors for CRT. Pediatric patients diagnosed with leukemia have multiple risk factors for VTE formation, such as the presence of hypercoagulable blast cells, the pro-thrombotic nature of the cancer itself, and treatment with steroids and L-asparaginase. Chen et al.^38^ and Jaffray et al.^4^ concluded that children with leukemia are more likely to develop CRT. Sepsis causes the coagulation mechanism to become fragile, which in turn activates the coagulation system and creates thrombosis.^72^ However, a study by Onyeama et al.^52^ showed that sepsis was significantly associated with a reduced incidence of CRT, and the exact mechanism is currently unknown.

The location of insertion and type of catheter are critical risk factors for CRT. The incidence of CRT is higher in femoral vein catheterizations compared to subclavian and jugular vein catheterizations in children, which is contrary to findings in adult patients.^73^ The femoral location is a larger vessel and allows placement of a larger size catheter. Femoral CVAD is prioritized in urgent and emergency situations. In such cases, the patients tend to be more critically ill and often immobilized, further exacerbating the low-flow state. In addition, there may be vein compression and kinking beneath the inguinal ligament with leg movement, which may increase the risk of CRT.^27^ PICC catheters provide a reliable medium to long-term route to intravenous therapy for children, but compared with other types of catheters, the risk of CRT is higher. We speculate that the long tunnel length and relatively large lumen size of the PICC, compared to the diameter of the vessel at the insertion site, may lead to increased blood flow obstruction.^52^ Additionally, patients with PICC may be more likely to be diagnosed with symptomatic VTE than tunneled lines (TLs) because PICC is often placed in smaller vessels and journeys through the arm or leg causing limb pain and swelling, whereas TLs are located in the chest.

The risk of CRT increases with the number of lumens. A possible explanation for this finding is that multilumen catheters tend to have larger catheter sizes and thus occupy more area within the vessel lumen, leading to obstruction of normal blood flow within the veins. The relationship between CRT and CLABSI is bidirectional. Following catheter insertion, a fibrin sheath forms around the catheter. Microorganisms, especially staphylococcus aureus, easily adhere to the fibrin sheaths, and may lead to CLABSI.^74^ Conversely, CLABSI can trigger inflammatory reactions, leading to further progression of thrombosis. CVAD duration is positively associated with the risk of CRT. Catheter placement may cause mechanical injury to the vein. As the indwelling duration increases, many damaged smooth muscle and endothelial cells become embedded within the fibrin, resulting in thrombus formation. In addition, prolonged indwelling increases the chance of platelet contact with the vessel lining, activating coagulation factors and thrombin, increasing the risk of thrombosis.^22^ Therefore, nurses should perform routine maintenance of the catheter in children who require long-term CVAD indwelling. The duration of CVAD should be monitored, the necessity of its indwelling should be assessed daily, and the catheter should be removed as early as possible while ensuring treatment.

As obstruction of venous blood flow from the CVAD is considered an essential causative mechanism for the development of VTE, a high ratio between catheter size and vein diameter could be a risk factor for CRT. The 2012 international guidelines on pediatric CVC insertion recommend that the ratio between the catheter’s external diameter and the cannulated vein’s diameter should not exceed 0.33.^75^ However, this suggestion is only based on expert opinions and currently lacks relevant clinical data support. Therefore, further research is still needed to verify it. Catheter dysfunction is mainly caused by small clots or fibrous sheaths wrapping around the tip of the catheter. Prolonged accumulation may lead to incomplete or complete blockage of blood vessels, becoming a gathering point for thrombosis.^74^ Journeycake et al. observed that the risk of VTE was highest in pediatric cancer patients with multiple episodes of catheter dysfunction.^76^ A study of pediatric brain tumor patients reported that VTE was more common in patients with catheter dysfunction.^77^ Thus, these studies and the current data support the need to consider catheter dysfunction as a possible risk factor for CRT and to design further screening and intervention studies for early identification and prevention of catheter dysfunction.

The rationale for studying the relationship between the insertion side of CVAD and the risk of CRT is based on the anatomy of the upper body venous system. The left brachiocephalic vein is longer and courses more horizontally than the right side, thus entering the superior vena cava at a sharper angle. The right jugular vein is the most direct and shortest route for the CVAD to enter the heart. By contrast, the CVAD located in the left jugular vein has a greater distance to the heart and passes through 2 angles in the venous system, which may cause endothelial damage and increase the likelihood of blood flow obstruction and venous wall adhesion.^26^ However, our meta-analysis did not find a statistically significant increase in the risk of CRT with left-sided placement compared to right-sided placement. The ideal location for the catheter tip is the junction of the superior vena cava and the right atrium. This location is preferred because of the higher blood flow rate, which may be protective against thrombosis.^43^ Currently, the pediatric literature on the effect of optimal tip position on CRT is scarce and inconclusive. In addition, catheter tips do not always remain in that position after initial placement. Therefore, tip movement should be a significant concern in pediatric patients, especially active, growing, and requiring long-term catheter use.

Providing renal replacement therapy is a lifelong task for pediatric end-stage renal disease (ESRD) patients. Although successful transplantation can be achieved even in young patients, the lifespan of the graft is limited. Consequently, many transplant recipients may be put back on dialysis as part of their ESRD treatment.^78^ CVC remains the main vascular access for hemodialysis in children. Long-term reliance on CVC is related to a high incidence of catheter dysfunction and failure. The frequent need for recurrent CVC placement in such patients leads to an elevated risk of central vein stenosis and CRT. Cardiac catheterization is also a possible risk factor for CRT. Appropriate anticoagulation is required during catheterization, without which the risk of thrombosis is up to 40%. However, the use of unfractionated heparin in pediatric patients is challenging because the coagulation system and heparin response are different from that of adults.^79^ There’s a need for further research to determine if children are receiving adequate doses of heparin during cardiac catheterization to prevent thrombosis without increasing the risk of bleeding complications. The incidence of VTE in adult patients who are chronically bedridden and braked is 3.59 times higher than in patients with normal activity levels.^80^ In critically ill or surgical children, mechanical ventilation is often performed in the early stages, requiring continuous use of multiple sedative or inotropic drugs to reduce cardiac load and protect pulmonary function. During sedation, the child is in a braked state, limb activity is reduced or even inactive, blood flow slows down, and blood stagnates in the veins, increasing the chance of platelet adhesion to the endothelium, which may increase the risk of CRT. Therefore, passive movements such as limb abduction, internal rotation, elbow flexion and elbow extension should be performed appropriately when the child’s condition permits.

Nutritional support is an important part of critical illness treatment, including enteral and parenteral nutrition (PN). CVAD is the supply channel for total parenteral nutrition (TPN), and some children may even need this method to provide calories for a long time. High glucose and calcium concentrations in PN are both possible triggers of CRT, and PN has been shown to upregulate the extrinsic coagulation cascade, especially with long-term use.^60^ Diamanti et al. reported that the incidence rate of TPN complicated with CRT was 20%.^81^ Mannitol or glycerol fructose are widely used as hypertonic drugs in clinical practice, which can increase plasma osmolality to dehydrate tissues after entering the body. At the same time, it may cause a cellular stress response, induce apoptosis, and can activate inflammatory cytokines and coagulation pathways to induce thrombosis. Jiang et al.^22^ found vasoactive drugs to be a risk factor for CRT. The possible reason is that vasoactive drugs can cause strong vasoconstriction, endothelial function damage or impairment, and promote fibrinogen synthesis. However, this is contrary to the findings of Marquez et al.^28^ and Faustino et al.^21^ Therefore, larger prospective studies are still needed to assess this risk factor more precisely.

The strengths of this study include the systematic identification of all relevant studies of risk factors for CRT in hospitalized children and the classification of risk factors into three categories, patient-related risk factors, CVAD-related risk factors, and treatment-related risk factors, to offer a logical progression of the possible causes of CRT in children. However, several limitations of this systematic review should be stated. Firstly, as most of the studies originate from Western countries, extrapolating these results to Eastern populations is questionable. Second, significant heterogeneity was encountered in our analysis, potentially stemming from variations in regimen, duration, population enrolled, and center setting, among other factors. This diversity necessitates a cautious interpretation of the results. In addition, only a few high-quality studies with a low risk of bias, and many of the studies suffer from significant sources of bias. Furthermore, the effect in many occasions was assessed by very few studies. Therefore, the evidence to support it is low, which needs to be validated in future studies. Finally, risk factors for CRT could not be made causal assertions since the majority of studies were retrospective.

## Conclusions

In conclusion, we have identified several critical factors that affect CRT, including D-dimer, location of insertion, type of catheter, number of lumens, catheter indwelling time, and CLABSI. Nevertheless, none of the included studies considered the impact of socio-demographic factors on CRT, such as parental education level, occupation, and family economic status. Therefore, larger sample sizes and well-designed prospective studies are still needed to clarify the predictors affecting CRT in the future. In addition, there is a lack of pediatric-specific CRT risk assessment tools, which need to be further developed and validated. Machine learning (ML), as a method for designing risk assessment models that help to efficiently explore and mine useful information, has been widely used in recent years to solve a variety of challenging medical problems. Likewise, the application of ML in CRT risk diagnosis may contribute to a more precise assessment. In clinical practice, it is necessary to take appropriate stratified preventive measures according to the level of CRT risk assessment of children, to improve the efficiency of clinical work, reduce the burden of clinical work, and minimize the occurrence of CRT under the premise of ensuring the safety of children.

## Supplementary information

Supplementary checklist

Supplemental Digital TableS1

Supplemental Digital TableS2

Supplemental Digital

## Data Availability

The datasets used and/or analyzed during the current study are available from the corresponding author upon reasonable request.
