# Supplementary material for: Risk factors and incidence of central venous access device-related thrombosis in hospitalized children: a systematic review and meta-analysis
Source: Pediatr Res. 2024 May 17;96(7):1568–93. doi: 10.1038/s41390-024-03225-0 (PMC11772251; doi:10.1038/s41390-024-03225-0)
Supplement: Supplementary file 4 — Supplemental Digital [file 41390_2024_3225_MOESM4_ESM.pdf]

Supplemental Digital: Database search strings

Pubmed (751)

(((((((("Child"[Mesh]) OR "Child, Preschool"[Mesh]) OR "Adolescent"[Mesh]) OR "Infant, Newborn"[Mesh]) OR "Infant"[Mesh]) OR "Pediatrics"[Mesh]) OR "Neonatology"[Mesh]) OR (children[Text Word] OR "preschool child"[Text Word] OR "children, preschool"[Text Word] OR "preschool children"[Text Word] OR adolescent*[Text Word] OR adolescence[Text Word] OR teen*[Text Word] OR teenager*[Text Word] OR youth*[Text Word] OR "female adolescent*"[Text Word] OR "male adolescent*"[Text Word] OR toddler[Text Word] OR "newborn infant*"[Text Word] OR newborn*[Text Word] OR neonate*[Text Word] OR infant[Text Word] OR baby[Text Word] OR neonatology[Text Word] OR child[Text Word] OR "child, preschool"[Text Word] OR "infant, newborn"[Text Word] OR pediatrics[Text Word] OR kid[Text Word] OR kids[Text Word])) AND ("central venous access device-related thrombosis"[Text Word] OR "CVAD-related thrombosis"[Text Word] OR CRT[Text Word] OR "central venous line-related thrombosis"[Text Word] OR "catheter-related thrombosis"[Text Word] OR "catheter-associated thrombosis"[Text Word] OR "catheter-related vascular thrombosis"[Text Word] OR "catheter-related venous thrombosis"[Text Word] OR "catheter-related deep venous thrombosis"[Text Word] OR "catheter-related venous thromboembolism"[Text Word] OR "catheter-related VTE"[Text Word] OR "catheter-related DVT"[Text Word] OR CRVT[Text Word] OR "central venous catheter-related thrombosis"[Text Word] OR "CVC-related thrombosis"[Text Word] OR "peripherally inserted central catheter-related thrombosis"[Text Word] OR "PICC-related thrombosis"[Text Word] OR "central venous catheter-related venous thrombosis"[Text Word] OR "CVC-related venous thrombosis"[Text Word] OR "peripherally inserted central catheter-related venous thrombosis"[Text Word] OR "PICC-related venous thrombosis"[Text Word] OR "CVC-related VTE"[Text Word] OR "PICC-related VTE"[Text Word] OR "central venous catheter-associated deep vein thrombosis"[Text Word] OR "CVC-associated DVT"[Text Word] OR

CADVT[Text Word] OR "PICC-associated DVT"[Text Word] OR "CVC-related complication"[Text Word] OR "PICC-related complication"[Text Word] OR "PORT-related complication"[Text Word])) AND (((((("Risk Factors"[Mesh]) OR "Risk Assessment"[Mesh]) OR "Protective Factors"[Mesh]) OR "Precipitating Factors"[Mesh]) OR "Causality"[Mesh]) OR (determinant*[Text Word] OR predictor*[Text Word] OR prediction[Text Word] OR indicator*[Text Word] OR causality[Text Word] OR causalities[Text Word] OR cause*[Text Word] OR factor*[Text Word] OR "clinical predictor*"[Text Word] OR "factor, risk"[Text Word] OR "risk factor*"[Text Word] OR "influencing factor*"[Text Word] OR "risk assessment*"[Text Word] OR "facilitating factor*"[Text Word] OR "hindering factor*"[Text Word] OR "protective factor*"[Text Word] OR "predictor variable*"[Text Word] OR "precipitating factor*"[Text Word] OR "enabling factor*"[Text Word]))

Web of science (901)

((TS=(children OR “preschool child” OR “children, preschool” OR “preschool children” OR adolescent* OR adolescence OR teen* OR teenager* OR youth* OR “female adolescent*” OR “male adolescent*” OR toddler OR “newborn infant*” OR newborn* OR neonate* OR infant OR baby OR neonatology OR child OR “child, preschool” OR “infant, newborn” OR pediatrics OR kid OR kids)) AND TS=(“central venous access device-related thrombosis” OR “CVAD-related thrombosis” OR CRT OR “central venous line-related thrombosis” OR “catheter-related thrombosis” OR “catheter-associated thrombosis” OR “catheter-related vascular thrombosis” OR “catheter-related venous thrombosis” OR “catheter-related deep venous thrombosis” OR “catheter-related venous thromboembolism” OR “catheter-related VTE” OR “catheter-related DVT” OR CRVT OR “central venous catheter-related thrombosis” OR “CVC-related thrombosis” OR “peripherally inserted central catheter-related thrombosis” OR “PICC-related thrombosis” OR “central venous catheter-related venous thrombosis” OR “CVC-related venous thrombosis” OR “peripherally inserted

central catheter-related venous thrombosis” OR “PICC-related venous thrombosis” OR “CVC-related VTE” OR “PICC-related VTE” OR “central venous catheter-associated deep vein thrombosis” OR “CVC-associated DVT” OR CADVT OR “PICC-associated DVT” OR “CVC-related complication” OR “PICC-related complication” OR “PORT-related complication”)) AND TS=(determinant* OR predictor* OR prediction OR indicator* OR causality OR causalities OR cause* OR factor* OR “clinical predictor*” OR “factor, risk” OR “risk factor*” OR “influencing factor*” OR “risk assessment*” OR “facilitating factor*” OR “hindering factor*” OR “protective factor*” OR “predictor variable*” OR “precipitating factor*” OR “enabling factor*”)

Cochrane (180)

#1MeSH descriptor: [Child] explode all trees

#2MeSH descriptor: [Child, Preschool] explode all trees

#3MeSH descriptor: [Adolescent] explode all trees

#4MeSH descriptor: [Infant, Newborn] explode all trees

#5MeSH descriptor: [Infant] explode all trees

#6MeSH descriptor: [Pediatrics] explode all trees

#7MeSH descriptor: [Neonatology] explode all trees

#8(children OR “preschool child” OR “children, preschool” OR “preschool children” OR adolescent* OR adolescence OR teen* OR teenager* OR youth* OR “female adolescent*” OR “male adolescent*” OR toddler OR “newborn infant*” OR newborn* OR neonate* OR infant OR baby OR neonatology OR child OR “child, preschool” OR “infant, newborn” OR pediatrics OR kid OR kids)

#9 #1 OR #2 OR #3 OR #4 OR #5 OR #6 OR #7 OR #8

#10(“central venous access device-related thrombosis” OR “CVAD-related thrombosis” OR CRT OR “central venous line-related thrombosis” OR “catheter-related thrombosis” OR “catheter-associated thrombosis” OR “catheter-related vascular thrombosis” OR “catheter-related venous thrombosis” OR

“catheter-related deep venous thrombosis” OR “catheter-related venous thromboembolism” OR “catheter-related VTE” OR “catheter-related DVT” OR CRVT OR “central venous catheter-related thrombosis” OR “CVC-related thrombosis” OR “peripherally inserted central catheter-related thrombosis” OR “PICC-related thrombosis” OR “central venous catheter-related venous thrombosis” OR “CVC-related venous thrombosis” OR “peripherally inserted central catheter-related venous thrombosis” OR “PICC-related venous thrombosis” OR “CVC-related VTE” OR “PICC-related VTE” OR “central venous catheter-associated deep vein thrombosis” OR “CVC-associated DVT” OR CADVT OR “PICC-associated DVT” OR “CVC-related complication” OR “PICC-related complication” OR “PORT-related complication”)

#11MeSH descriptor: [Risk Factors] explode all trees

#12MeSH descriptor: [Risk Assessment] explode all trees

#13MeSH descriptor: [Protective Factors] explode all trees

#14MeSH descriptor: [Precipitating Factors] explode all trees

#15MeSH descriptor: [Causality] explode all trees

#16(determinant* OR predictor* OR prediction OR indicator* OR causality OR causalities OR cause* OR factor* OR “clinical predictor*” OR “factor, risk” OR “risk factor*” OR “influencing factor*” OR “risk assessment*” OR “facilitating factor*” OR “hindering factor*” OR “protective factor*” OR “predictor variable*” OR “precipitating factor*” OR “enabling factor*”)

#17#11 OR #12 OR #13 OR #14 OR #15 OR #16

#18#9 AND #10 AND #17

Embase (927)

#1'child'/exp

#2'preschool child'/exp

#3'adolescent'/exp

#4'newborn'/exp

#5'infant'/exp

#6'pediatrics'/exp

#7'neonatology'/exp

#8children:ti,ab,kw OR 'preschool child':ti,ab,kw OR 'children, preschool':ti,ab,kw OR 'preschool children':ti,ab,kw OR adolescent*:ti,ab,kw OR adolescence:ti,ab,kw OR teen*:ti,ab,kw OR teenager*:ti,ab,kw OR youth*:ti,ab,kw OR 'female adolescent*':ti,ab,kw OR 'male adolescent*':ti,ab,kw OR toddler:ti,ab,kw OR 'newborn infant*':ti,ab,kw OR newborn*:ti,ab,kw OR neonate*:ti,ab,kw OR infant:ti,ab,kw OR baby:ti,ab,kw OR neonatology:ti,ab,kw OR child:ti,ab,kw OR 'child, preschool':ti,ab,kw OR 'infant, newborn':ti,ab,kw OR pediatrics:ti,ab,kw OR kid:ti,ab,kw OR kids:ti,ab,kw

#9 #1 OR #2 OR #3 OR #4 OR #5 OR #6 OR #7 OR #8

#10'central venous access device-related thrombosis':ti,ab,kw OR 'cvad-related thrombosis':ti,ab,kw OR crt:ti,ab,kw OR 'central venous line-related thrombosis':ti,ab,kw OR 'catheter-related thrombosis':ti,ab,kw OR 'catheter-associated thrombosis':ti,ab,kw OR 'catheter-related vascular thrombosis':ti,ab,kw OR 'catheter-related venous thrombosis':ti,ab,kw OR 'catheter-related deep venous thrombosis':ti,ab,kw OR 'catheter-related venous thromboembolism':ti,ab,kw OR 'catheter-related vte':ti,ab,kw OR 'catheter-related dvt':ti,ab,kw OR crvt:ti,ab,kw OR 'central venous catheter-related thrombosis':ti,ab,kw OR 'cvc-related thrombosis':ti,ab,kw OR 'peripherally inserted central catheter-related thrombosis':ti,ab,kw OR 'picc-related thrombosis':ti,ab,kw OR 'central venous catheter-related venous thrombosis':ti,ab,kw OR 'cvc-related venous thrombosis':ti,ab,kw OR 'peripherally inserted central catheter-related venous thrombosis':ti,ab,kw OR 'picc-related venous thrombosis':ti,ab,kw OR 'cvc-related vte':ti,ab,kw OR 'picc-related vte':ti,ab,kw OR 'central venous catheter-associated deep vein thrombosis':ti,ab,kw OR 'cvc-associated dvt':ti,ab,kw OR cadvt:ti,ab,kw OR 'picc-associated dvt':ti,ab,kw OR 'cvc-related complication':ti,ab,kw OR 'picc-related complication':ti,ab,kw OR 'port-related complication':ti,ab,kw

#11'risk factor'/exp

#12'risk assessment'/exp

#13'causality'/exp

#14determinant*:ti,ab,kw OR predictor*:ti,ab,kw OR prediction:ti,ab,kw OR indicator*:ti,ab,kw OR causality:ti,ab,kw OR causalities:ti,ab,kw OR cause*:ti,ab,kw OR factor*:ti,ab,kw OR 'clinical predictor*':ti,ab,kw OR 'factor, risk':ti,ab,kw OR 'risk factor*':ti,ab,kw OR 'influencing factor*':ti,ab,kw OR 'risk assessment*':ti,ab,kw OR 'facilitating factor*':ti,ab,kw OR 'hindering factor*':ti,ab,kw OR 'protective factor*':ti,ab,kw OR 'predictor variable*':ti,ab,kw OR 'precipitating factor*':ti,ab,kw OR 'enabling factor*':ti,ab,kw

#15 #11 OR #12 OR #13 OR #14

#16 #9 AND #10 AND #15

Scopus (1011)

(TITLE-ABS-KEY (children OR "preschool child" OR "children, preschool" OR "preschool children" OR adolescent* OR adolescence OR teen* OR teenager* OR youth* OR "female adolescent*" OR "male adolescent*" OR toddler OR "newborn infant*" OR newborn* OR neonate* OR infant OR baby OR neonatology OR child OR "child, preschool" OR "infant, newborn" OR pediatrics OR kid OR kids) AND TITLE-ABS-KEY ("central venous access device-related thrombosis" OR "CVAD-related thrombosis" OR crt OR "central venous line-related thrombosis" OR "catheter-related thrombosis" OR "catheter-associated thrombosis" OR "catheter-related vascular thrombosis" OR "catheter-related venous thrombosis" OR "catheter-related deep venous thrombosis" OR "catheter-related venous thromboembolism" OR "catheter-related VTE" OR "catheter-related DVT" OR crvt OR "central venous catheter-related thrombosis" OR "CVC-related thrombosis" OR "peripherally inserted central catheter-related thrombosis" OR "PICC-related thrombosis" OR "central venous catheter-related venous thrombosis" OR "CVC-related venous thrombosis" OR "peripherally inserted central catheter-related venous thrombosis" OR "PICC-related venous thrombosis" OR "CVC-related VTE"

OR "PICC-related VTE" OR "central venous catheter-associated deep vein thrombosis" OR "CVC-associated DVT" OR cadvt OR "PICC-associated DVT" OR "CVC-related complication" OR "PICC-related complication" OR "PORT-related complication") AND TITLE-ABS-KEY (determinant* OR predictor* OR prediction OR indicator* OR causality OR causalities OR cause* OR factor* OR "clinical predictor*" OR "factor, risk" OR "risk factor*" OR "influencing factor*" OR "risk assessment*" OR "facilitating factor*" OR "hindering factor*" OR "protective factor*" OR "predictor variable*" OR "precipitating factor*" OR "enabling factor*"))

Sinomed (114)

("儿童"[全部字段:智能] OR "儿科"[全部字段:智能] OR "学龄前儿童"[全部字段:智能] OR "学龄期儿童"[全部字段:智能] OR "青年"[全部字段:智能] OR "青少年"[全部字段:智能] OR "青少年儿童"[全部字段:智能] OR "新生儿"[全部字段:智能] OR "早产儿"[全部字段:智能]) AND (("中心静脉导管相关性深静脉血栓"[全部字段:智能] OR "CVC 相关性 DVT"[全部字段:智能] OR "CADVT"[全部字段:智能] OR "PICC 相关性 DVT"[全部字段:智能] OR "CVC 相关性并发症"[全部字段:智能] OR "PICC 相关性并发症"[全部字段:智能] OR "PORT 相关性并发症"[全部字段:智能]) OR ("导管相关性 VTE"[全部字段:智能] OR "CRVT"[全部字段:智能] OR "CVC 相关性血栓"[全部字段:智能] OR "外周静脉置入中心静脉导管相关性血栓"[全部字段:智能] OR "PICC 相关性血栓"[全部字段:智能] OR "输液港相关性血栓"[全部字段:智能] OR "中心静脉导管相关静脉血栓形成"[全部字段:智能] OR "CVC 相关性 VTE"[全部字段:智能] OR "PICC 相关性 VTE"[全部字段:智能]) OR ("中心静脉输液装置相关性血栓"[全部字段:智能] OR "CVAD 相关性血栓"[全部字段:智能] OR "CRT"[全部字段:智能] OR "中心静脉导管相关性血栓"[全部字段:

智能] OR "导管相关性血栓"[全部字段:智能] OR "导管相关血栓"[全部字段:智能] OR "导管相关性静脉血栓"[全部字段:智能] OR "导管相关性深静脉血栓"[全部字段:智能] OR "导管相关性静脉血栓栓塞"[全部字段:智能])) AND ("危险因素"[全部字段:智能] OR "风险因素"[全部字段:智能] OR "影响因素"[全部字段:智能] OR "预测因素"[全部字段:智能] OR "因素"[全部字段:智能] OR "保护因素"[全部字段:智能] OR "阻碍因素"[全部字段:智能] OR "促进因素"[全部字段:智能])

CNKI (116)

((主题=(儿童 + 儿科 + 学龄前儿童 + 学龄期儿童 + 青年 + 青少年 + 青少年儿童 + 新生儿 + 早产儿)) AND 主题=(中心静脉输液装置相关性血栓 + CVAD 相关性血栓 + CRT + 中心静脉导管相关性血栓 + 导管相关性血栓 + 导管相关血栓 + 导管相关性静脉血栓 + 导管相关性深静脉血栓 + 导管相关性静脉血栓栓塞 + 导管相关性 VTE + CRVT + CVC 相关性血栓 + 外周静脉置入中心静脉导管相关性血栓 + PICC 相关性血栓 + 输液港相关性血栓 + 中心静脉导管相关静脉血栓形成 + CVC 相关性 VTE + PICC 相关性 VTE + 中心静脉导管相关性深静脉血栓 + CVC 相关性 DVT + CADVT + PICC 相关性 DVT + CVC 相关性并发症 + PICC 相关性并发症 + PORT 相关性并发症)) AND 主题=(危险因素 + 风险因素 + 影响因素 + 预测因素 + 因素 + 保护因素 + 阻碍因素 + 促进因素)

Wanfang (193)

((主题=(儿童 or 儿科 or 学龄前儿童 or 学龄期儿童 or 青年 or 青少年 or 青

少年儿童 or 新生儿 or 早产儿)) AND 主题=(中心静脉输液装置相关性血栓 or CVAD 相关性血栓 or CRT or 中心静脉导管相关性血栓 or 导管相关性血栓 or 导管相关血栓 or 导管相关性静脉血栓 or 导管相关性深静脉血栓 or 导管相关性静脉血栓栓塞 or 导管相关性 VTE or CRVT or CVC 相关性血栓 or 外周静脉置入中心静脉导管相关性血栓 or PICC 相关性血栓 or 输液港相关性血栓 or 中心静脉导管相关静脉血栓形成 or CVC 相关性 VTE or PICC 相关性 VTE or 中心静脉导管相关性深静脉血栓 or CVC 相关性 DVT or CADVT or PICC 相关性 DVT or CVC 相关性并发症 or PICC 相关性并发症 or PORT 相关性并发症)) AND 主题=(危险因素 or 风险因素 or 影响因素 or 预测因素 or 因素 or 保护因素 or 阻碍因素 or 促进因素)

Box 1.

Defining the Strength of a Risk Factor

Defining the Strength of a Risk Factor

Definite

All low and moderate risk of bias studies positive (at least three studies)

Majority (more than 50%) low and moderate risk of bias studies positive (at least five studies)

Likely

All low and moderate risk of bias studies positive (two studies)

Majority (more than 50%) low and moderate risk of bias studies positive (2–4 studies)

Unclear

All low and moderate risk of bias studies positive (one study)

Low and moderate risk of bias studies show mixed or conflicting results

A majority (more than 50%) of studies negative but at least one low or moderate risk of bias study positive

Not a risk factor

No low or moderate risk of bias studies positive

Supplemental Digital: Fig.S1-S7

Study	log[Odds Ratio]	SE	Weight	Odds Ratio IV, Random, 95% CI
**Incidence of CRT in children**				
Badheka 2021	-2.14921536	0.13208518	2.1%	0.12 [0.09, 0.15]
Beck1988	-1.49752	0.26829354	2.1%	0.22 [0.13, 0.38]
Charny 2018	-2.26868354	0.24759378	2.1%	0.10 [0.06, 0.17]
Chen 2016	-1.56397554	0.25919773	2.1%	0.21 [0.13, 0.35]
Chojnacka 2022	-3.7968927	0.25278949	2.1%	0.02 [0.01, 0.04]
Deng 2020	-1.81773496	0.24734306	2.1%	0.16 [0.10, 0.26]
Derderian 2019	-4.2024513	0.15929209	2.1%	0.01 [0.01, 0.02]
Diamond 2018	-2.12823171	0.47308511	2.0%	0.12 [0.05, 0.30]
Dubois 2007	-2.27212589	0.23485025	2.1%	0.10 [0.07, 0.16]
Faustino 2013	-1.67006253	0.27251551	2.1%	0.19 [0.11, 0.32]
Faustino 2015	-0.2602831	0.21877011	2.1%	0.77 [0.50, 1.18]
Gnannt 2018	-3.12217337	0.1065293	2.2%	0.04 [0.04, 0.05]
Gray 2012	-1.51512723	0.14258215	2.1%	0.22 [0.17, 0.29]
Haddad 2014	-2.12200116	0.12739256	2.1%	0.12 [0.09, 0.15]
Jaffray 2020	-2.86402293	0.10604289	2.2%	0.06 [0.05, 0.07]
Jiang 2022	-1.22820512	0.1561819	2.1%	0.29 [0.22, 0.40]
Jones 2019	-1.27046255	0.20005482	2.1%	0.28 [0.19, 0.42]
Kim 2022	-0.45783309	0.22949124	2.1%	0.63 [0.40, 0.99]
Lambert 2019	-3.78552564	0.24527259	2.1%	0.02 [0.01, 0.04]
Li 2021	-1.70281198	0.06474638	2.2%	0.18 [0.16, 0.21]
Li 2022	-1.01504721	0.09285845	2.2%	0.36 [0.30, 0.43]
Longo 2021	-2.39789527	0.22268089	2.1%	0.09 [0.06, 0.14]
Lovett 2023	-0.85348983	0.24899152	2.1%	0.43 [0.26, 0.69]
MacLean 2018	-2.91777073	0.19402265	2.1%	0.05 [0.04, 0.08]
Male 2003	-0.65805586	0.22877915	2.1%	0.52 [0.33, 0.81]
Male 2005	-1.87545849	0.23434658	2.1%	0.15 [0.10, 0.24]
Marquez 2016	-0.83372913	0.16451336	2.1%	0.43 [0.31, 0.60]
McLaughlin 2019	-2.7131653	0.28640027	2.1%	0.07 [0.04, 0.12]
Menéndez 2016	-0.34294475	0.13938491	2.1%	0.71 [0.54, 0.93]
Noonan 2018	-3.62966009	0.12109767	2.2%	0.03 [0.02, 0.03]
Onyeama 2018	-2.30258509	0.24720662	2.1%	0.10 [0.06, 0.16]
Östlund 2019	-0.8315495	0.14975888	2.1%	0.44 [0.32, 0.58]
Patel 2020	-5.18685361	0.05020234	2.2%	0.01 [0.01, 0.01]
Pei 2016	-2.63905733	0.3912304	2.1%	0.07 [0.03, 0.15]
Rooden 2005	-1.95683922	0.29629823	2.1%	0.14 [0.08, 0.25]
Shah 2015	-4.081085	0.1280681	2.1%	0.02 [0.01, 0.02]
Shin 2017	-1.01780826	0.0682809	2.2%	0.36 [0.32, 0.41]
Smitherman 2015	-3.07449211	0.17047426	2.1%	0.05 [0.03, 0.06]
Sol 2015	-2.23084119	0.29186905	2.1%	0.11 [0.06, 0.19]
Steen 2019	-2.49364033	0.13781558	2.1%	0.08 [0.06, 0.11]
Tran 2018	-4.58306116	0.02511178	2.2%	0.01 [0.01, 0.01]
Verheij 2018	-3.64880123	0.27071629	2.1%	0.03 [0.02, 0.04]
Wang 2021	-3.84565826	0.23185435	2.1%	0.02 [0.01, 0.03]
Wei 2017	-0.92233305	0.20579413	2.1%	0.40 [0.27, 0.60]
Wisecup 2015	-2.89613291	0.09187977	2.2%	0.06 [0.05, 0.07]
Zeng 2020	-1.05954482	0.12441188	2.2%	0.35 [0.27, 0.44]
Zhu 2022	-6.07238599	0.37839995	2.1%	0.00 [0.00, 0.00]
**Total (95% CI)**			**100.0%**	**0.10 [0.06, 0.17]**

Heterogeneity: $Tau^2 = 2.94$; $Chi^2 = 9451.31$, df = 46 ($P < 0.00001$); $I^2 = 100\%$

Test for overall effect: Z = 9.09 ($P < 0.00001$)

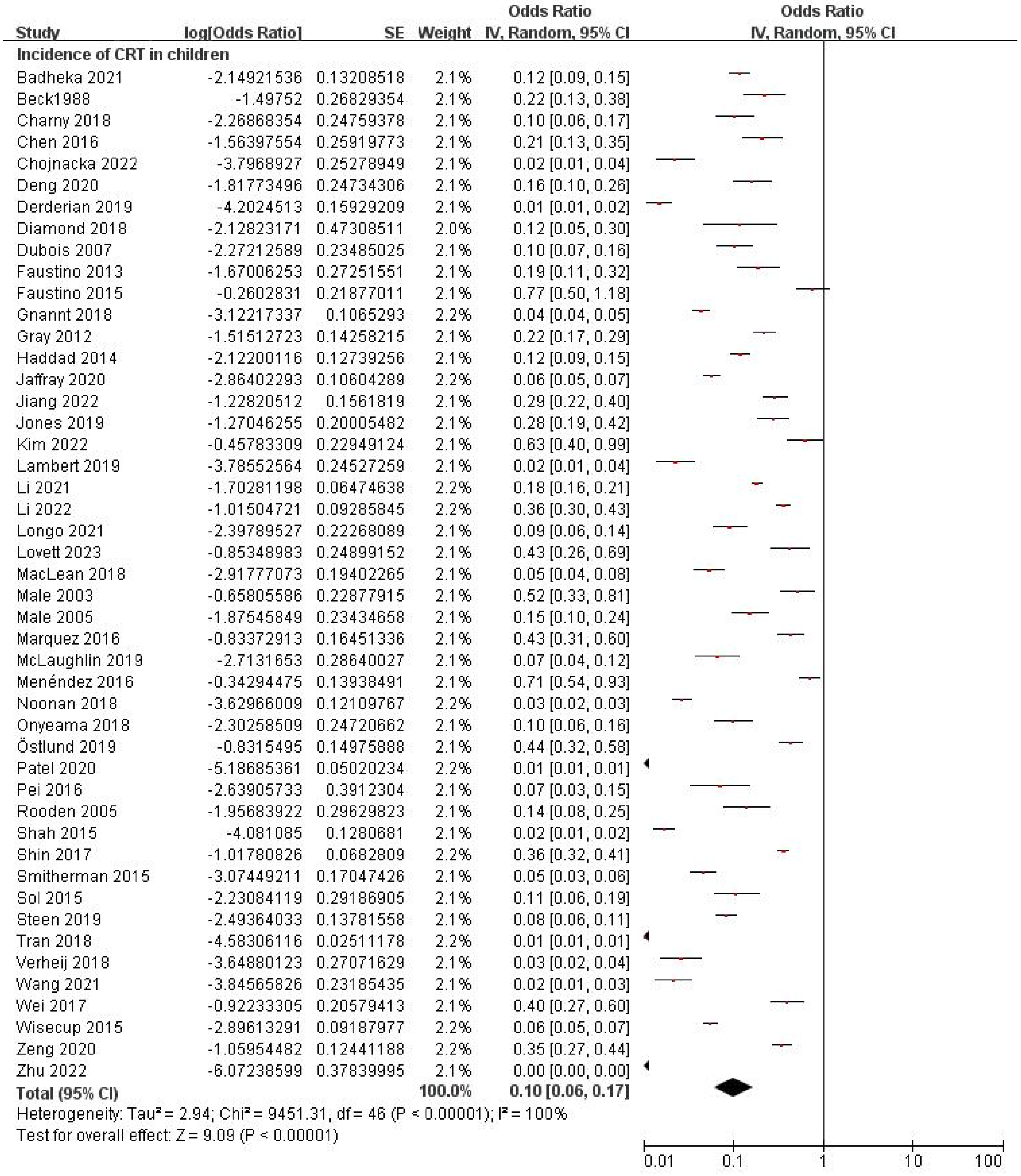

Fig. S1 Meta integration of CRT rate in children

Study	log[Odds Ratio]	SE	Weight	Odds Ratio IV, Random, 95% CI
**Incidence of CRT in famale children**				
Beck1988	-1.18958407	0.43166585	4.5%	0.30 [0.13, 0.71]
Chen 2016	-1.54044504	0.36731544	4.6%	0.21 [0.10, 0.44]
Deng 2020	-1.92990981	0.35670736	4.6%	0.15 [0.07, 0.29]
Dubois 2007	-1.82685079	0.28796323	4.6%	0.16 [0.09, 0.28]
Faustino 2013	-1.75785792	0.4842342	4.5%	0.17 [0.07, 0.45]
Gnannt 2018	-3.30771349	0.18286252	4.7%	0.04 [0.03, 0.05]
Kim 2022	-0.30538165	0.35222173	4.6%	0.74 [0.37, 1.47]
Lambert 2019	-4.45725006	0.5028902	4.4%	0.01 [0.00, 0.03]
Li 2021	-1.82336481	0.1032269	4.7%	0.16 [0.13, 0.20]
Lovett 2023	-2.19722458	0.74535599	4.1%	0.11 [0.03, 0.48]
Male 2003	-0.31845373	0.32856437	4.6%	0.73 [0.38, 1.38]
Male 2005	-1.64222774	0.31537655	4.6%	0.19 [0.10, 0.36]
Menéndez 2016	-0.50209194	0.18680746	4.7%	0.61 [0.42, 0.87]
Onyeama 2018	-2.09323486	0.35328422	4.6%	0.12 [0.06, 0.25]
Östlund 2019	-1.12986483	0.25100531	4.7%	0.32 [0.20, 0.53]
Pei 2016	-2.42774824	0.60228324	4.3%	0.09 [0.03, 0.29]
Smitherman 2015	-2.81054948	0.22468729	4.7%	0.06 [0.04, 0.09]
Sol 2015	-2.67414865	0.73108328	4.2%	0.07 [0.02, 0.29]
Tran 2018	-4.58275415	0.03745788	4.7%	0.01 [0.01, 0.01]
Verheij 2018	-4.57471098	0.58031867	4.4%	0.01 [0.00, 0.03]
Wei 2017	-0.75030559	0.294281	4.6%	0.47 [0.27, 0.84]
Zeng 2020	-1.04982212	0.1963961	4.7%	0.35 [0.24, 0.51]
**Total (95% CI)**			**100.0%**	**0.13 [0.06, 0.30]**

Heterogeneity: Tau² = 3.87; Chi² = 2014.76, df = 21 (P < 0.00001); I² = 99%

Test for overall effect: Z = 4.74 (P < 0.00001)

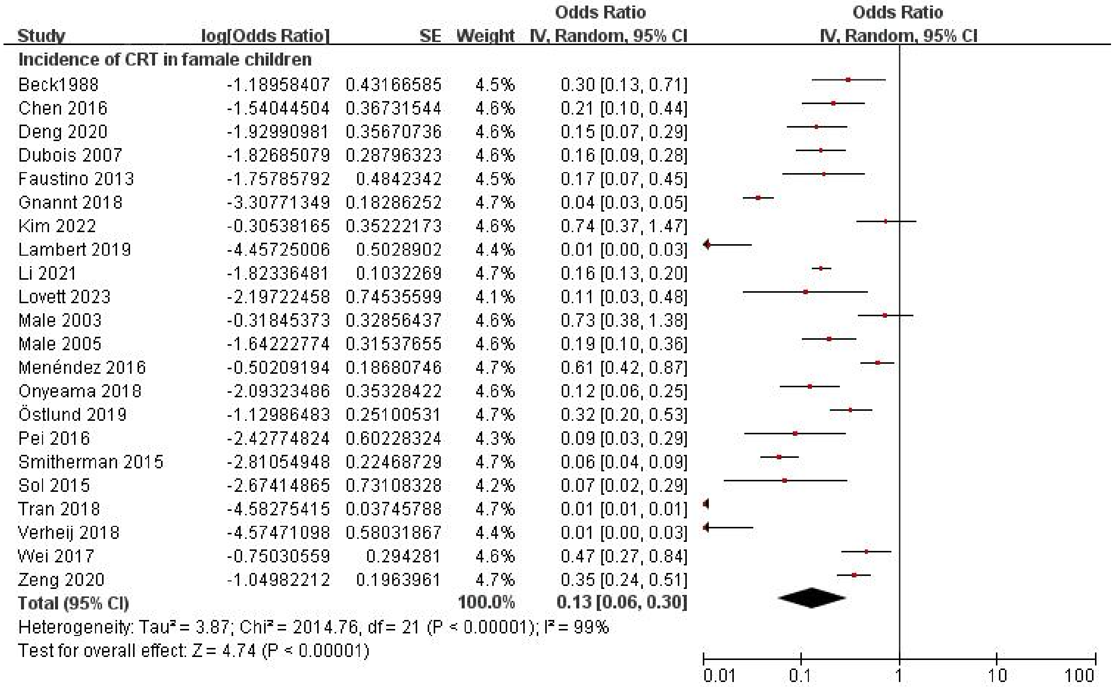

Fig. S2 Meta integration of CRT rate in female children

Study	log[Odds Ratio]	SE	Weight	Odds Ratio IV, Random, 95% CI
**Incidence of CRT in male children**				
Beck1988	-2.10413415	0.47369847	4.4%	0.12 [0.05, 0.31]
Chen 2016	-1.58696506	0.36583929	4.5%	0.20 [0.10, 0.42]
Deng 2020	-1.70474809	0.34377582	4.5%	0.18 [0.09, 0.36]
Dubois 2007	-2.44716632	0.34745719	4.5%	0.09 [0.04, 0.17]
Faustino 2013	-1.62745642	0.32979726	4.5%	0.20 [0.10, 0.37]
Gnannt 2018	-3.10964309	0.15069526	4.6%	0.04 [0.03, 0.06]
Kim 2022	-0.56798404	0.30357349	4.5%	0.57 [0.31, 1.03]
Lambert 2019	-3.43646552	0.28177708	4.6%	0.03 [0.02, 0.06]
Li 2021	-1.61864396	0.08322153	4.6%	0.20 [0.17, 0.23]
Lovett 2023	-0.5389965	0.27458482	4.6%	0.58 [0.34, 1.00]
Male 2003	-0.96141117	0.32609024	4.5%	0.38 [0.20, 0.72]
Male 2005	-2.12026354	0.35276684	4.5%	0.12 [0.06, 0.24]
Menéndez 2016	-0.8774509	0.18360423	4.6%	0.42 [0.29, 0.60]
Onyeama 2018	-2.47560426	0.34706902	4.5%	0.08 [0.04, 0.17]
Östlund 2019	-0.64551913	0.18828419	4.6%	0.52 [0.36, 0.76]
Pei 2016	-2.77258872	0.5153882	4.4%	0.06 [0.02, 0.17]
Smitherman 2015	-3.35573501	0.26266375	4.6%	0.03 [0.02, 0.06]
Sol 2015	-2.47443535	0.36813899	4.5%	0.08 [0.04, 0.17]
Tran 2018	-4.5833117	0.03384339	4.7%	0.01 [0.01, 0.01]
Verheij 2018	-3.46289095	0.30619944	4.5%	0.03 [0.02, 0.06]
Wei 2017	-1.07755888	0.28944187	4.6%	0.34 [0.19, 0.60]
Zeng 2020	-1.06603612	0.16078957	4.6%	0.34 [0.25, 0.47]
**Total (95% CI)**			**100.0%**	**0.13 [0.06, 0.30]**

Heterogeneity: Tau² = 3.76; Chi² = 2658.03, df = 21 (P < 0.00001); I² = 99%

Test for overall effect: Z = 4.85 (P < 0.00001)

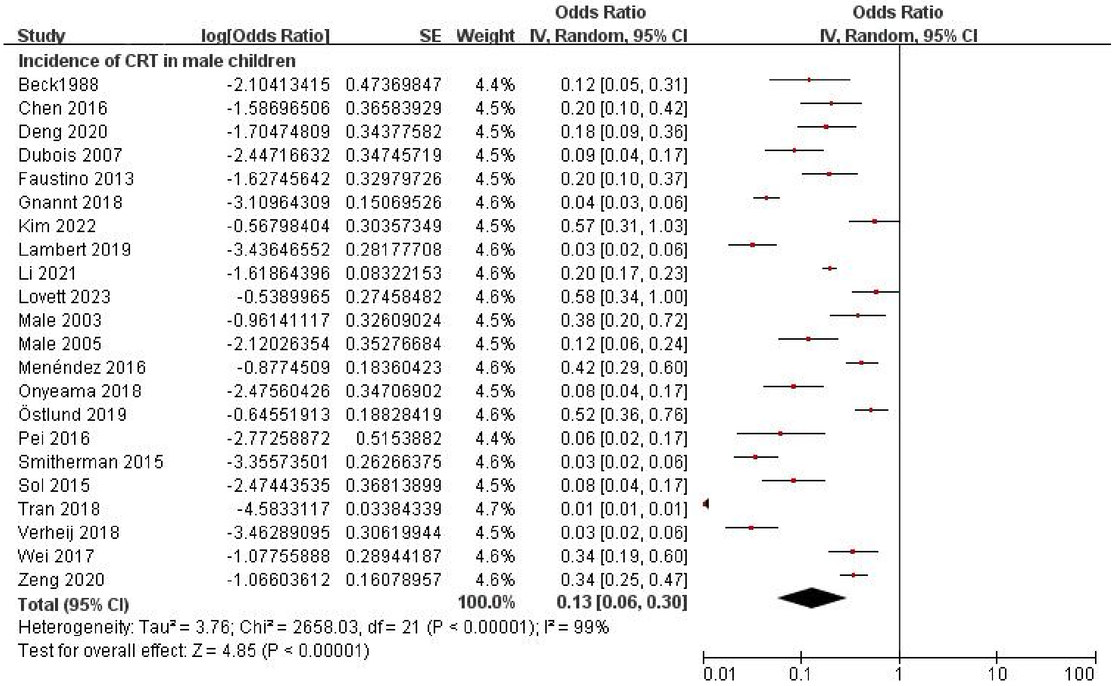

Fig. S3 Meta integration of CRT rate in male children

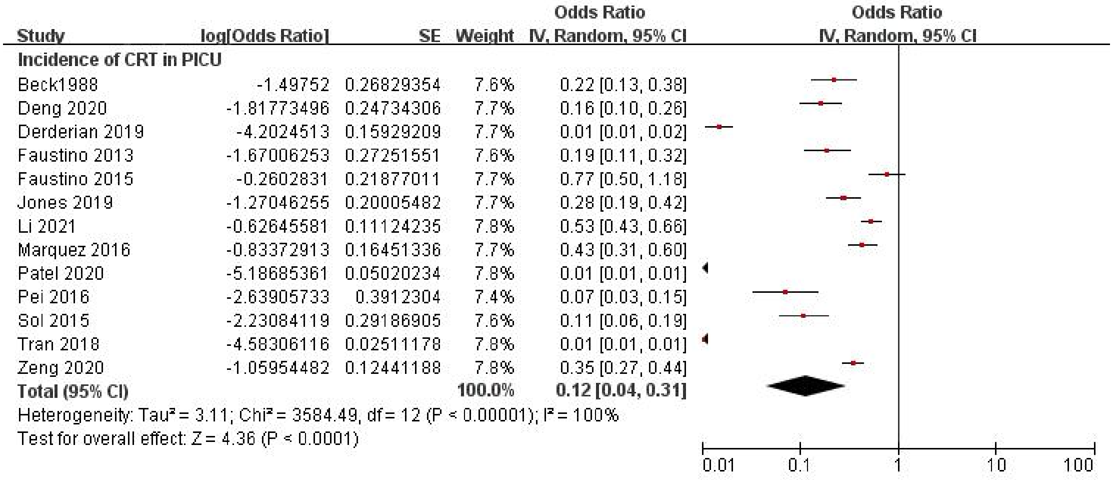

Fig. S4 Meta integration of CRT rate in PICU

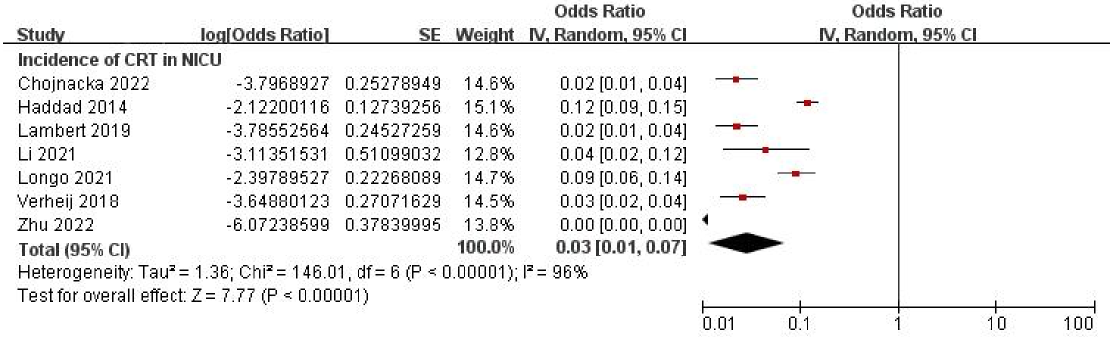

Fig. S5 Meta integration of CRT rate in NICU

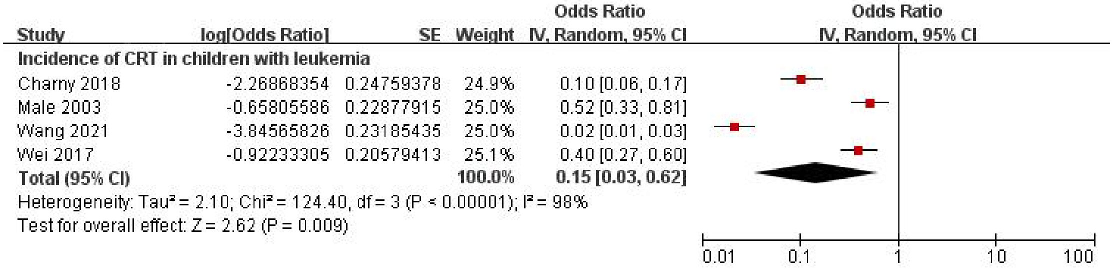

Fig. S6 Meta integration of CRT rate in children with leukemia

	Selection of participants	Confounding variables	Measurement of exposure	Blinding of outcome assessments	Incomplete outcome data	Selective outcome reporting	Summary risk of bias
Badheka 2021	+	+	+	+	?	+	?
Beck1988	+	+	+	+	+	+	+
Charny 2018	+	+	+	+	+	+	+
Chen 2016	+	?	+	+	?	?	?
Chojnacka 2022	+	+	+	+	?	?	?
Deng 2020	+	+	+	+	?	?	?
Derderian 2019	+	+	+	+	?	+	?
Diamond 2018	+	−	+	+	?	+	−
Dubois 2007	+	+	+	+	+	+	+
Faustino 2013	+	+	+	+	?	+	?
Faustino 2015	+	+	+	+	+	+	+
Gnannt 2018	+	+	+	?	+	+	?
Gray 2012	+	?	+	?	?	+	?
Haddad 2014	+	−	+	+	+	+	−
Jaffray 2020	+	+	+	+	+	+	+
Jiang 2022	+	+	+	+	?	+	?
Jones 2019	+	+	+	+	+	+	+
Kim 2022	+	+	+	+	+	?	?
Lambert 2019	+	+	+	?	+	?	?
Li 2021	+	+	+	+	+	?	?
Li 2022	+	+	+	?	+	+	?
Longo 2021	+	+	+	+	?	?	?
Lovett 2023	+	+	+	?	+	+	?
MacLean 2018	?	?	+	+	+	+	?
Male 2003	?	+	+	+	?	+	?
Male 2005	−	+	+	+	+	+	−
Marquez 2016	+	+	+	+	+	+	+
McLaughlin 2019	+	−	+	?	+	+	−
Menéndez 2016	+	+	+	?	+	?	?
Noonan 2018	+	?	+	+	+	+	?
Onyeama 2018	+	+	+	+	+	?	?
Östlund 2019	+	+	+	+	+	+	+
Patel 2020	?	+	+	+	+	+	?
Pei 2016	+	?	+	+	+	?	?
Rooden 2005	+	?	+	?	+	+	?
Shah 2015	+	+	+	?	?	?	?
Shin 2017	+	+	+	?	+	+	?
Smitherman 2015	+	+	+	+	?	+	?
Sol 2015	+	+	+	?	+	+	?
Steen 2019	+	+	+	+	+	+	+
Tran 2018	+	+	+	?	+	+	?
Verheij 2018	+	+	+	+	+	?	?
Wang 2021	+	?	+	?	+	+	?
Wei 2017	+	+	+	+	?	+	?
Wisecup 2015	+	+	+	?	+	?	?
Zeng 2020	+	+	+	+	?	?	?
Zhu 2022	+	+	+	+	?	+	?

Fig. S7 Quality evaluation of each study
